# MXenes from MAX phases: synthesis, hybridization, and advances in supercapacitor applications

**DOI:** 10.1039/d5ra00271k

**Published:** 2025-03-24

**Authors:** Tamal K. Paul, Md. Abdul Khaleque, Md. Romzan Ali, Mohamed Aly Saad Aly, Md. Sadek Bacchu, Saidur Rahman, Md. Zaved H. Khan

**Affiliations:** a Laboratory of Nano-Bio and Advanced Materials Engineering (NAME), Jashore University of Science and Technology Jashore 7408 Bangladesh mohamed.alysaadaly@ece.gatech.edu zaved.khan@just.edu.bd; b Department of Chemical Engineering, Jashore University of Science and Technology Jashore 7408 Bangladesh; c School of Electrical and Computer Engineering, Georgia Institute of Technology Atlanta GA 30332 USA; d Department of Electrical and Computer Engineering at Georgia Tech Shenzhen Institute (GTSI) Shenzhen Guangdong 518052 China; e Research Centre for Nano-Materials and Energy Technology, School of Engineering and Technology, Sunway University Bandar Sunway Malaysia; f Department of Engineering, Lancaster University Lancaster UK

## Abstract

MXenes, which are essentially 2D layered structures composed of transition metal carbides and nitrides obtained from MAX phases, have gained substantial interest in the field of energy storage, especially for their potential as electrodes in supercapacitors due to their unique properties such as high electrical conductivity, large surface area, and tunable surface chemistry that enable efficient charge storage. However, their practical implementation is hindered by challenges like self-restacking, oxidation, and restricted ion transport within the layered structure. This review focuses on the synthesis process of MXenes from MAX phases, highlighting the different etching techniques employed and how they significantly influence the resulting MXene structure and subsequent electrochemical performance. It further highlights the hybridization of MXenes with carbon-based materials, conducting polymers, and metal oxides to enhance charge storage capacity, cyclic stability, and ion diffusion. The influence of dimensional structuring (1D, 2D, and 3D architectures) on electrochemical performance is critically analyzed, showcasing their role in optimizing electrolyte accessibility and energy density. Additionally, the review highlights that while MXene-based supercapacitors have seen significant advancements in terms of energy storage efficiency through various material combinations and fabrication techniques, key challenges like large-scale production, long-term stability, and compatibility with electrolytes still need to be addressed. Future research should prioritize developing scalable synthesis methods, optimizing hybrid material interactions, and investigating new electrolyte systems to fully realize the potential of MXene-based supercapacitors for commercial applications. This comprehensive review provides a roadmap for researchers aiming to bridge the gap between laboratory research and commercial supercapacitor applications.

## Abstract 2

MXenes, which are essentially 2D layered structures composed of transition metal carbides and nitrides obtained from MAX phases.

## Graphical abstract

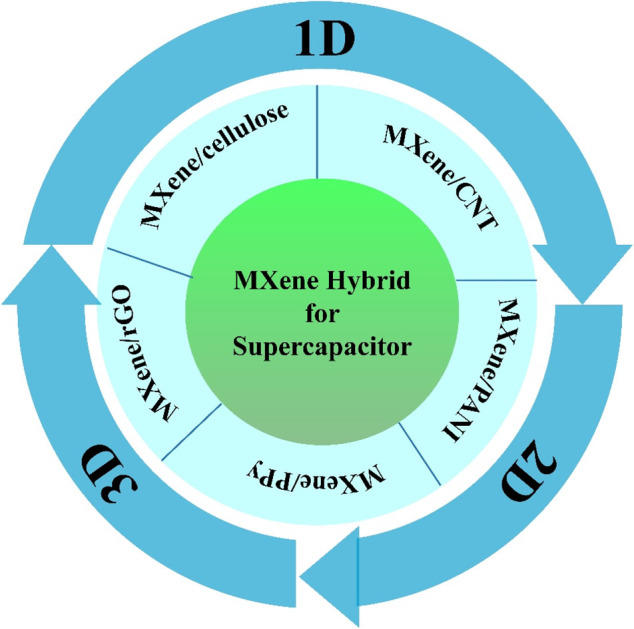

## 1 Introduction

Currently, environmental problems are considered the most concerning issues in the growing usage of fossil fuel-based products. To mitigate the adverse effect of fossil fuels on the environment, alternative environmentally friendly energy sources such as geothermal energy, wind energy, solar energy, and hydropower are extensively investigated. Although these sources are environmentally friendly, it largely depends on nature to consistently supply the energy demands. Therefore, in order to reduce the use of fossil fuels and lower the dependence on these natural sources, a new energy storage device needs to be explored.^1^ Therefore, scientists are trying to develop supercapacitors, parallel plate capacitors that can store energy as batteries,^2,3^ as a new alternative type of energy storage device. Supercapacitors have fast charge and discharge rates with excellent cyclic capability and high power density.^4,5^ Electrode materials play a crucial role for supercapacitor applications. It has been reported that electrode materials prepared from carbon based materials (CNT, rGO, *etc.*),^6^ transition metal based materials^7^ and conductive polymers^8^ have been used in supercapacitor-related applications. Among these active materials, transition metal nitrides and carbides known as MXene, discovered by Naguib *et. al.*,^9^ are extensively used for supercapacitor applications due to their high electrical conductivity, fast ion diffusion and excellent hydrophilic characteristics.^10^

MXene can be synthesized from MAX phases, where M refers to transition metal such as V, Sc, Zr, Cr, Ti and Mo; a represents metal element such as Sn, Ga, Ti, Ge, In, Al, Si, Cd, P, As, S; and X indicates carbon (C) or nitrogen (N) atoms.^11^ MXene can be produced by etching the element “A” from the MAX phase using different etching agents such as HF, H_3_PO_4_, NaOH or LiF.^11^ The general formula of MXene is denoted by M_*n*+1_X_*n*_T_*x*_, where M symbolizes a transition metal, X represents C or N atoms, T denotes the surface terminating groups such as –OH, –O, –F, introduced during etching process.^12^ This negative surface groups of MXene makes it an excellent substrate for hybridization with other materials.^13^ Furthermore, due to the inherent conductivity and the potentiality of charge transfer provided by the transition metal M changeable oxidation number, MXene exhibits exceptional electrochemical properties, and therefore, it is well suited for use in supercapacitor applications.^12,14^ Although MXene has the potential characteristics to fabricate excellent electrode materials for supercapacitor applications, it has some major issues that may reduce electrochemical performance. During the fabrication of MXene from MAX phases, a wide variety of negative functional groups are induced on the MXene surface, which is why, aggregation occurs in MXene suspension due to the van der walls interaction between these polar groups.^15^ Furthermore, the structural stability of pure MXene-based electrodes during the cyclic performance may be hampered due to the restacking nature of MXene.^16^ Additionally, Ti_3_C_2_T_*x*_ may be partially oxidized by oxygen or water molecules into the nonconductive titanium dioxide (TiO_2_), decreasing the redox reaction active sites and raising the charge transfer impedance.^17^ Scientists are trying to solve these flaws by preparing MXene-based hybrid materials to enhance their capacitive characteristics. To overcome these drawbacks, scientists took the advantages of wide surface terminating groups of MXenes, that allows MXene materials to interact with other active material. This interaction increases the interlayer spacing of MXene by avoiding the aggregation problem for which ion transport between the MXene based hybrids is enhanced. Therefore, the capacitive behavior of MXene based hybrid structure is improved. The most promising hybridization strategies include: (i) MXene/carbon-based hybrids: carbon nanotubes (CNTs), graphene, and activated carbon can be incorporated with MXenes to enhance conductivity, prevent restacking, and increase surface area for improved ion diffusion. (ii) MXene/conducting polymer hybrids: polyaniline (PANI), polypyrrole (PPy), and PEDOT:PSS provide pseudocapacitance, boosting energy storage capacity while maintaining flexibility and mechanical stability. (iii) MXene/metal compound hybrids: transition metal oxides (TMOs) and transition metal dichalcogenides (TMDs) improve charge storage due to their redox activity, increasing the overall capacitance and energy density. For example, Wang *et al.*^18^ prepared MXene/PDA film where PDA acted as an interlayer spacer, reducing self-stacking during cycling. In this hybrid structure, Ti made strong bonds with oxygen atoms in polydopamine whereas dopamine formed hydrogen bonds with surface functional groups, ensuring the stability of the structure.^18^ Liu *et al.* fabricated MXene/cellulose hybrid where cellulose increased the interlayer space of MXene and also ensured the good mechanical (124.6 MPa) and electromagnetic properties (36 dB).^19^ For using additive materials with MXene nanosheets, enhanced electrochemical performance has been achieved which is extensively discussed by many reports to enlighten the authors about the recent research of MXene in supercapacitors. For example, Luo *et al.* reported the application of MXene/conducting polymers (PPy, PANI, PEDOT:PSS) composites in the research of supercapacitors by discussing the preparation MXene/conducting polymers electrodes and their uses in supercapacitors.^20^ Thomas *et al.* highlighted the supercapacitor applications of MXene hybrids with carbonaceous materials, conducting polymers, transition metal dichalcogenides (TMDs), transition metal oxides (TMOs), *etc.*, thorough their fundamental properties, synthesis tactics and etching procedures comprising various kind of MXenes.^21^ Besides the interaction of MXene and other active materials for excellent supercapacitor applications, some other factors, like electrolytes, dimensional structure of hybrid materials, fabrication technique of hybrid materials, are very important to enhance the electrochemical performance. These factors greatly influence capacitive performance. However, there are some reports whereas these factors are highlighted. For example, while the research conducted by Zang *et al.* primarily investigated ways to improve the capacitance of a material by manipulating its surface, creating films, and combining it with other materials (creating a composite), they did not delve deeply into other factors that could also significantly impact capacitance, such as the type of electrolyte used, the shape and size of the material (dimensional structures), and the specific methods used to create the material (fabrication techniques).^4^ Orangi *et al.* elaborately discussed the fabrication process of MXenes based electrode materials for energy storage applications, however, they did not extensively analyze the influence of multidimensional structural design, interlayer spacing, and ion diffusion on capacitive performance.^22^ Hu *et al.* shed light on the MXene-based supercapacitor performance focusing on structure, design, surface chemistry, electrode architecture and composites of MXenes, however, future challenges (aggregation, oxidation, scalability) and the possible solution for these hurdles weren't discussed.^12^ While the review reported by Panda *et al.* thoroughly examined how factors like MXene sheet size, shape, electrode architecture, and electrolyte type impact the performance of MXene-based supercapacitors, it notably lacked a comprehensive analysis of how the multidimensional structural design (1D, 2D, and 3D) of MXene materials specifically influences ion transport and the overall capacitive performance within the device.^23^ Among all these factors, interlayer multidimensional structure of MXene hybrid materials (1D, 2D, 3D) has also a great influence in capacitive performance, because electrolyte ion transportation path largely depends on it which can affect the electrochemical performance. Hu *et al.* discussed the progress on MXene symmetric supercapacitor focusing on 1D, 2D, 3D structures.^12^

Herein, the influence of multidimensional structural design of MXene hybridized materials for capacitive performance is elaborately discussed. To the best of the authors' knowledge this is the first report on the influence of the dimensional structure of MXene hybrid materials in supercapacitor applications. Moreover, this review systematically discusses the procedure of MXene synthesis from MAX phases, the preparation strategy of MXene-based hybrid materials and their multidimensional structural analysis in supercapacitors. The effect of interlayer spacing ion diffusion and the electrochemical performance of multidimensional MXene hybrids are also analyzed extensively. Finally, an overall guideline is provided to tackle the challenges of preparing MXene-based hybrid materials for next-generation supercapacitor applications.

## 2 Synthesis of MXenes

### 2.1 MAX phases to MXene

The protocol of MAX phase etching attracted a great deal of attention among scientific communities because of the great demand of using MXene in materials development research. In “Top-down” selective etching strategies, MAX phase is converted to MXene by breaking the bonds between ‘M’ and ‘A’. In this procedure, the etching reaction is significantly sensitive to air and moisture (<1 ppm H_2_O; <5 ppm O_2_).^24^ The etching agents are categorized as acids (HF, H_3_PO_4_), alkali (NaOH), fluoride salt + HCl (LiF, KF, NH_4_F), molten salt (LiF + KF, CdBr_2_, ZnCl_2_), NH_4_HF_2_ and others. Among these, fluoride salt + HCl (LiF, KF, NH_4_F) affects the multi-layered MXenes synthesis when intercalation fabrication method is used. That is, the etching agent mixture causes the interlayer space to expand by increasing lattice parameter and weaken interflake interactions.^25^ A summary on carbide, nitride, and carbonitride precursors etching events is represented in Table 1. In contrast, the delamination process is only suitable for a few layer-flakes exfoliation of MXenes. It is a mechanical exfoliation of MXenes, and it is comparatively challenging than intercalation.

**Table 1:** A comparison of 2D MXenes etching from their MAX phases

MAX phase	MXene	Etching agent	Temperature (°C)	Time (hour)	Yield (%)	Ref.
Ti_2_AlC	Ti_2_CT_*x*_	10% HF	Room temp.	10	80	26
V_2_AlC	V_2_CT_*x*_	50% HF	Room temp.	92	60	27
Nb_2_CT_*x*_	Nb_2_CT_*x*_	50% HF	Room temp.	90	100	28
Ti_2_AlN	Ti_2_NT_*x*_	KF + HCl	Room temp.	24	N/A	29
Ti_3_AlC_2_	Ti_3_C_2_T_*x*_	50% HF	Room temp.	2	100	9
(Ti_0.5_Nb_0.5_)_2_AlC	(Ti_0.5_Nb_0.5_)_2_CT_*x*_	51% HF	Room temp.	28	80	30
(V_0.5_Cr_0.5_)_3_AlC_2_	(V_0.5_Cr_0.5_)_3_C_2_T_*x*_	50% HF		69	N/A	
Ta_4_AlC_3_	Ta_4_C_3_T_*x*_	50% HF		72	90	
Nb_4_AlC_3_	Nb_4_C_3_T_*x*_	50% HF	Room temp.	96	77	31
Mo_2_TiAlC_2_	Mo_2_TiC_2_T_*x*_	50% HF	Room temp.	48	100	32
Mo_2_TiAlC_3_	Mo_2_TiC_3_T_*x*_	50% HF	55	90		
(Mo_2/3_Y_1/3_)_2_AlC	Mo_4/3_CT_*x*_	48% HF	Room temp.	60	N/A	33
		10% HF	Room temp.	72	N/A	33
Mo_2_TiAlC_2_	Mo_2_TiC_2_T_*x*_	48–51% HF	Room temp.	48	N/A	32
Mo_2_Ti_2_AlC_3_	Mo_2_Ti_2_C_3_T_*x*_	48–51% HF	55	90	N/A	32
(W_2/3_Sc_1/3_)_2_AlC	W_4/3_CT_*x*_	48% HF	Room temp.	30	N/A	34
Zr_3_Al_3_C_5_	Zr_3_C_2_T_*x*_	50% HF	Room temp.	60	N/A	35
Hf_3_[Al(Si)]_4_C_6_	Hf_3_C_2_T_*x*_	35% HF	Room temp.	60	N/A	36
Ti_2_AlC	Ti_2_CT_*x*_	0.9 M LiF + 6 M HCl	40	15	N/A	37
Mo_2_Ga_2_C	Mo_2_CT_*x*_	3 M LiF + 12 M HCl	35	384	N/A	38
Mo_2_Ga_2_C	Mo_2_CT_*x*_	NH_4_Cl + HCl	140–180	24	N/A	39
Nb_2_AlC	Nb_2_CT_*x*_	0.75 g NaBF_4_ + 37% HCl	180	15–35	N/A	40
V_2_AlC	V_2_CT_*x*_	2 g LiF + 40 M HCl	90	48	N/A	41
V_2_AlC	V_2_CT_*x*_	1.5 g NaF + HCl	90	120		42
V_2_AlC	V_2_CT_*x*_	LiF + HCl	90	120	N/A	43
V_2_AlC	V_2_CT_*x*_	2 g NaF + 1.24 g LiF + 4.48 g KF + 40 ml HCl	90	72	N/A	44
Ti_3_AlC_2_	Ti_3_C_2_T_*x*_	0.75 g NaBF_4_ + 37% HCl	180	8–32	N/A	40
Ti_3_AlC_2_	Ti_3_C_2_T_*x*_	1 g LiF + 6 M HCl	35	24	N/A	45
Ti_3_AlC_2_	Ti_3_C_2_T_*x*_	3 M LiF + 6 M HCl	40	45	100	46
Ti_3_AlCN	Ti_3_CNT_*x*_	0.66 g LiF + 6 M HCl	35	12	N/A	33
(Nb_0.8_Zr_0.2_)_4_AlC_3_	(Nb_0.8_Zr_0.2_)_4_C_3_T_*x*_	LiF +12 M HCl	50	168	N/A	47
(W_2/3_Sc_1/3_)_2_AlC	W_4/3_CT_*x*_	4 g LiF + 12 M HCl	35	48	N/A	34
Ti_3_AlC_2_	Ti_3_C_2_T_*x*_	1 M NH_4_HF_2_	80	12	N/A	48
Ti_3_AlC_2_	Ti_3_C_2_T_*x*_	NH_4_F	150	24	N/A	49
Ti_4_AlN_3_	Ti_4_N_3_T_*x*_	59% KF + 29% LiF + 12% NaF	550	0.5	N/A	50
Ti_3_AlC_2_	Ti_3_C_2_T_*x*_	2.07 g LiF + 3.35 g NaF + 7.52 g KF	30, 40, 50, 60	12, 24, 48	N/A	51
Ti_3_AlC_2_	Ti_3_C_2_T_*x*_	SnF_2_ (1 : 6)	550	6	N/A	52
Ti_4_AlN_3_	Ti_4_N_3_T_*x*_	KF + LiF + NaF	550	0.5	N/A	29

#### 2.1.1 HF solution etching

**Fig. 1:**
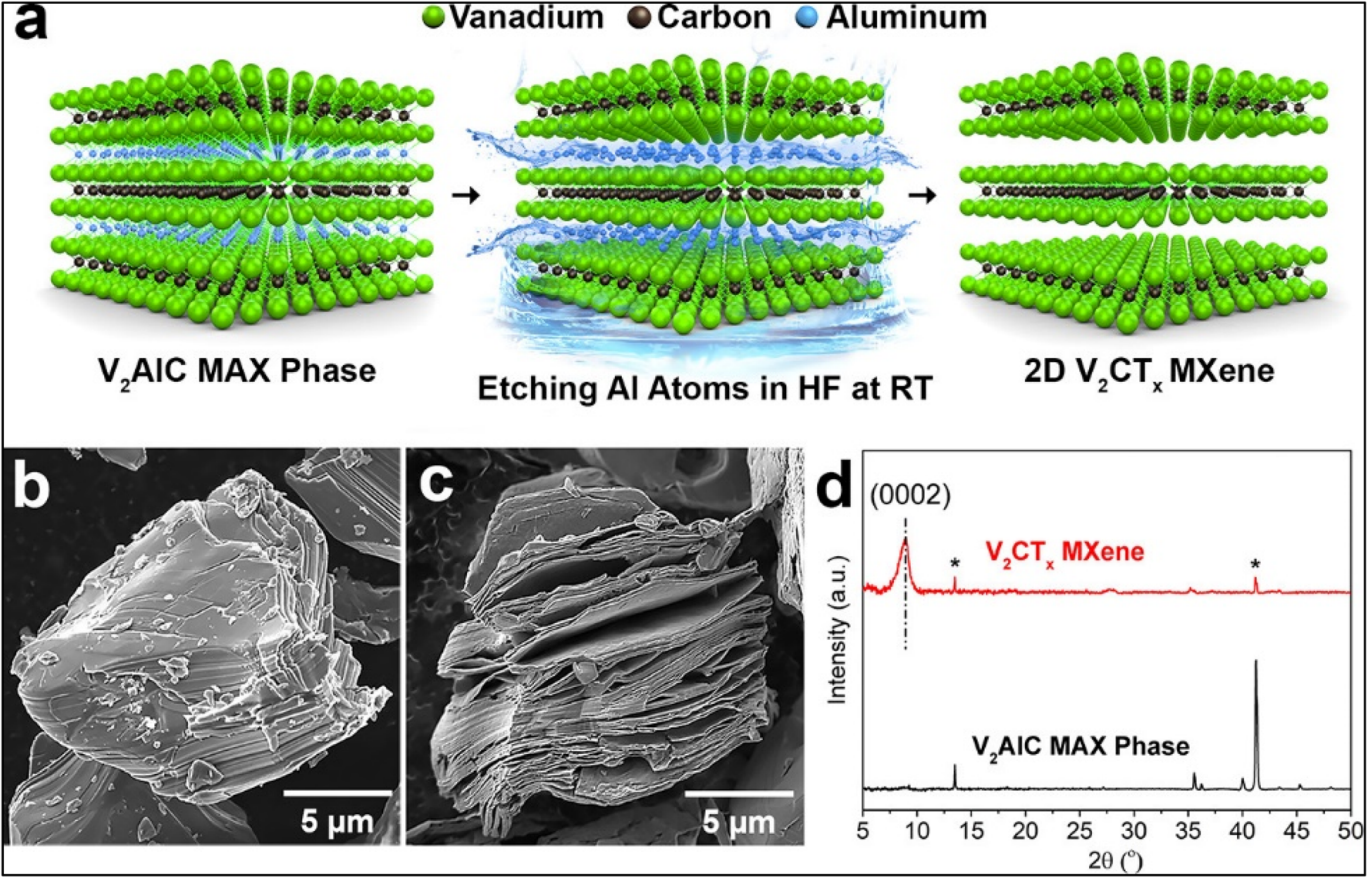
(a) Schematic representation of V_2_CT_*x*_ synthesis from V_2_AlC; SEM images of (b) V_2_AlC phase and (c) V_2_CT_*x*_. (d) XRD patterns of V_2_AlC and V_2_CT_*x*_, (*) denotes residual V_2_AlC phase.^27^

M. Naguib and coworkers first used 50% concentrated hydrogen fluoride (HF) etching agent for the synthesis of Ti_3_C_2_T_*x*_ from Ti_3_AlC_2_ at room temperature for 2 hours.^9^ In this study, successful etching was confirmed by the shifted main peak of XRD pattern of Ti_3_C_2_T_*x*_ and Ti_3_AlC_2_. Another 50% hydrofluoric acid (HF) treated MXenes was synthesized at room temperature with magnetic stirring at 200 rpm for 96 hours. The etched MXenes was washed several times with centrifugation at 4500 rpm reaching a pH level of 4 (ref. 27) and further was coined as multilayer MXenes. Here, a few layers of V_2_CT_*x*_ were synthesized using 1-methyl-2-pyrolidinone treatment and the inter sheet space was formed by intercalation treatment of tetrabutylammonium hydroxide (TBAOH). The explanation of HF etching phase transition is represented in Fig. 1. The XRD pattern proved the MXenes phase formation before and after the HF etching. Moreover, HF etching-based MXenes synthesis research were carried out by incorporating transition metal carbide nanosheets such as Ti_2_CT_*x*_,^53^ Nb_2_CT_*x*_,^28^ (Ti_0.5_Nb_0.5_)_2_CT_*x*_,^54^ (V_0.5_Cr_0.5_)_3_C_2_T_*x*_,^54^ Ta_4_C_3_T_*x*_,^54^ Nb_4_C_3_T_*x*_,^55^ Mo_2_TiC_2_T_*x*_,^32^ Mo_2_Ti_2_C_3_T_*x*_,^32^ W_4/3_CT_*x*_,^34^ Zr_3_C_2_T_*x*_,^35^ Hf_3_C_2_T_*x*_.^36^

#### 2.1.2 Fluoride and HCl mixture etching

**Fig. 2:**
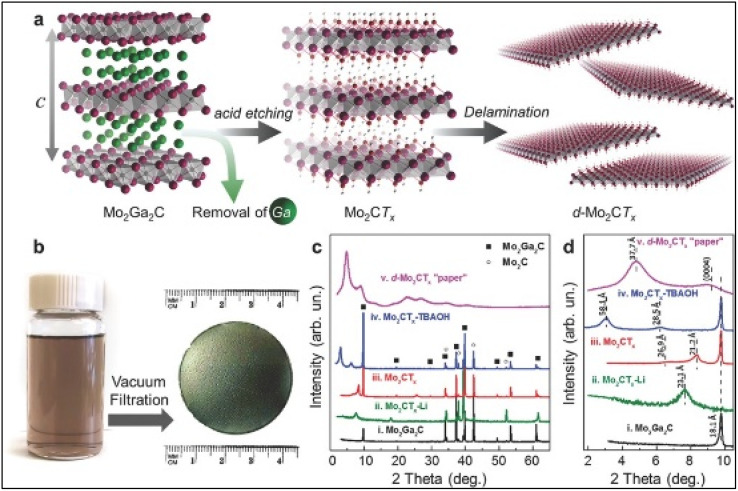
(a) Schematic representation of etching and delamination of Mo_2_Ga_2_C MAX phase; (b) digital images of delamination and filtration; (c) XRD patterns of (i) Mo_2_Ga_2_C (black), (ii) Mo_2_CT_*x*_–Li (green), (iii) Mo_2_CT_*x*_ (red), (iv) Mo_2_CT_*x*_ intercalated by TBAOH (blue) and (v) paper (purple), (d) same XRD patterns of (c) with the focus on the 2*θ* range of 2–10.5°.^38^

Metal nitride (Ti_2_N) etching is difficult for higher formation energy of Ti_2_AlN. Here, selective etching and intercalation are achieved by soaking Ti_2_AlN in a mixture of potassium fluoride (KF) and hydrochloric acid (HCl). Thereafter, exfoliation of Ti_2_N is done in DMSO to obtain few layer of Ti_2_NT_*x*_ flakes.^29^ Additionally, Halim *et al.*, reported a research work for large scale production of molybdenum carbide (Mo_2_CT_*x*_) MXene by selectively etching gallium from Mo_2_Ga_2_C precursor.^38^ In this study, etching was done by using a mixture of 3 M of LiF and 12 M of HCl, next, the intercalation process was continued by using TBAOH and finally delamination was completed.^38^ The synthesized Mo_2_CT_*x*_ was heat treated in a temperature range of 300–10 K to behave like a semiconductor, conversely, it behaved like metal. The synthesis procedures (etching, delamination and filtration) and the characterization results of this work are represented in Fig. 2.

Furthermore, fluoride salt and HCl etching protocol was comparatively suitable to synthesize multilayer flakes than other processes. There were different kinds of MXene synthesized and reported such as Ti_2_CT_*x*_,^37^ Nb_2_CT_*x*_,^56^ V_2_CT_*x*_,^41^ V_2_CT_*x*_,^42^ V_2_CT_*x*_,^43^ V_2_CT_*x*_,^44^ Ti_3_C_2_T_*x*_,^40^ Ti_3_C_2_T_*x*_,^45^ Ti_3_C_2_T_*x*_,^46^ Ti_3_CNT_*x*_,^33^ (Nb_0.8_Zr_0.2_)4C_3_T_*x*_,^47^ W_4/3_CT_*x*_.^34^

#### 2.1.3 Salt based etching

**Fig. 3:**
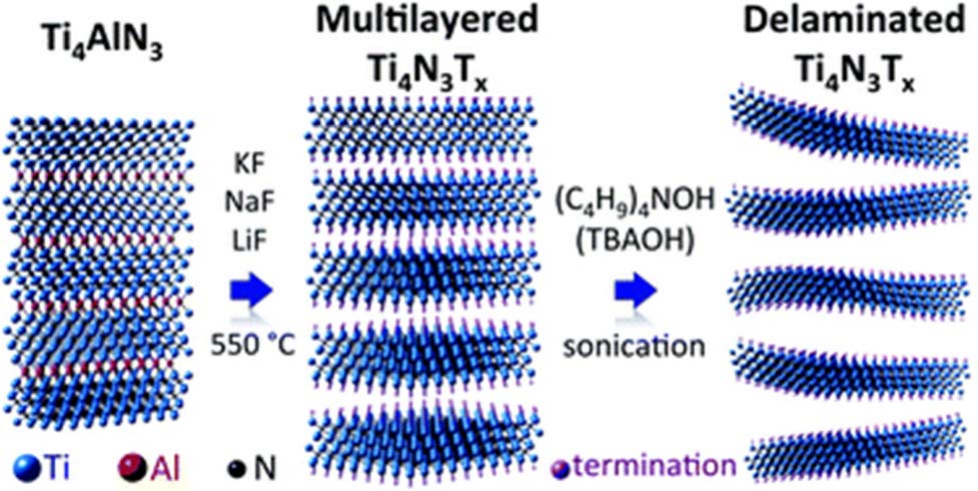
Schematic of molten salt synthesis of Ti_4_N_3_T_*x*_ from Ti_4_AlN_3_ at 550 °C and delamination by TBAOH.

Water dispersible Ti_3_C_2_T_*z*_ MXene was synthesized without the use of HF and lacked the –OH terminal group.^52^ Here, molten salt (SnF_2_) was used as a selective etchant to synthesize Ti_3_C_2_T_*z*_ from Ti_3_AlC_2_ MAX precursor. During etching, AlF_3_ was formed, and Sn remained as a byproduct that was etched by stirring and agitations. This was the first reported molten salt etching-based research work. Soundiraraju *et al.*^29^ were the first research work to report on the two-dimensional transition metal nitride, Ti_4_N_3_-based MXenes from Ti_4_AlN_3_.^53^ In this work, ternary eutectic composition, a mixture of salt KF, LiF, and NaF was maintained at 550 °C for 30 minutes with a heating incremental of 10 °C per minute.^29^ Furthermore, TBAOH was used in delamination for synthesis of few flakes. The etching and delamination process of this work is represented in Fig. 3. Moreover, major salt-based etching was performed at higher temperature at which the salt or mixture of salt solution can be melted. There are numerous research work reporting the synthesis of 2D MXenes, including Ti_3_C_2_T_*x*_,^48^ NH_4_F,^49^ KF + LiF + NaF,^50^ LiF + NaF + KF,^51^ SnF_2_ (1 : 6),^52^ KF + LiF + NaF.^29^

#### 2.1.4 Other etching methods

Although there are a variety of etching methods, only a few number studies was reported. The non-conventional etching strategies are electrochemical etching,^57^ hydrochloric acid etching,^58^ and alkaline etching with high temperature hydrothermal approach.^57^ Electrochemical etching involves flowing an electric current in an electrolyte solution to remove specific atomic layers from MAX phases, offering precise control over the etching process. Hydrochloric acid (HCl) etching is a simpler chemical approach where HCl selectively dissolves certain elements, though it may be less efficient than fluoride-based etching. Alkaline hydrothermal etching utilizes a heated alkaline solution under high pressure to break bonds in MAX phases, making it particularly useful for obtaining stable MXenes without strong acids. Compared to traditional HF etching, these methods can reduce safety hazards, improve structural control, and enhance environmental friendliness. However, each approach has limitations, such as slower reaction rates or incomplete etching, which researchers continue to refine for large-scale applications.

## 3 Possible hybridization of MXenes for supercapacitor application

MXene can be considered one of the best electrode materials for supercapacitor application. Lukatskaya *et al.* found that macroporous multilayered MXene (Ti_3_C_2_T_*x*_) film handled up to 210 F g^−1^ at 10 V s^−1^ of scan rate.^59^ However, it has been reported that freestanding individual MXene electrodes suffers from restacking and oxidation (in contact with oxygen and water) problems,^4^ for which reason intercalation of MXene with other materials is very necessary. In the following section, the possible hybridization of MXene materials with their preparation process and capacitive behavior are highlighted.

### 3.1 MXene/CNT hybrid

Carbon nanotube (CNT) possesses excellent electrical, thermal and mechanical properties. This increases the potential of utilizing CNT in developing promising materials in various applications such as wearable electronic devices, sensors, supercapacitors.^60^ Laser ablation, chemical vapor deposition (CVD) and arc discharge are the most commonly used methods to synthesis CNT. It is worth mentioning that as-prepared CNT may possess metallic impurities.^61^ Furthermore, CNT may be aggregated in colloidal suspension because of the van der Waals interaction between the sidewalls of CNT.^62^ These issues restrict the practical application of individual CNT. Thus, incorporating MXene material with CNT can be a possible solution. A hybrid material composed of MXene and Carbon Nanotubes (CNTs) overcomes the individual limitations of each component, exhibiting superior electrical and mechanical properties, a larger surface area, and high pore volume, making it a highly promising candidate for supercapacitor applications; researchers like Yu *et al.* have extensively explored the diverse applications of MXene/CNT hybrids, including various fabrication methods and structural architectures to optimize their performance across different applications.^63^ Here, the preparation process is summarized first, and then an elaborate discussion is made regarding the supercapacitor applications of multidimensional MXene/CNT hybrid material.

#### 3.1.1 Preparation process of MXene/CNT hybrid materials

Carbon nanotube (CNT) can perform dual activity of MXene/CNT hybrids for supercapacitor application. It can solve the aggregation problem of MXene, and further, it increases the interlayer spacing of MXene sheets, which effectively transfers electrolyte ions during the charge–discharge process, enhancing the electrochemical performance of MXene/CNT hybrids.

To synthesize MXene/CNT hybrid material, mainly two approaches are involved: the integration of CNT and MXene with chemically reactive (chemical) and without chemically reactive (physical) process. Preparing MXene and CNT hybrid material without chemically reactive process is an easy technique that involves different techniques like mechanical mixing,^64^ co-dispersion and self-assembly.^65^ Mechanical Mixing is the frequently used technique involving ultrasonication of a certain amount of MXene and CNT dispersion followed by vacuum filtration to prepare a thin film. Yan *et al.* prepared MXene/CNT hybrid material by ultrasonic stirring of MXene and CNT colloidal suspension followed by filtering the mixed dispersion.^64^ Regarding the self-assembly method, Guo *et al.* developed MXene/CNT composite material by taking the advantage of electrostatic interaction between MXene and CNT.^66^ The terminating group (–OH, –F, –O *etc.*) of MXene makes it a highly negative charged particle, ensuring strong electrochemical interaction with positively charged CNT-polyethyleneimine.^66^ There are many chemically reactive process involved in preparing MXene/CNT hybrid material such as *in situ* technique,^67^ thermal treatment,^68^ microwave process^69^ and hydrothermal process.^70^ Regarding the chemical process, it may need high energy consumption, like 800 °C for thermal treatment,^68^ which makes this process unsuitable for scalable production. On the other hand, mechanical mixing, self-assembly, co-dispersion methods are the easiest and most widely used techniques for the fabrication of MXene/CNT hybrids and also, in this regard the hybridized materials provide superior mechanical strength due to the hydrogen bonding between the materials.

The as-prepared MXene/CNT hybrid material by the above mentioned techniques can be formed into one-dimension (1D), two-dimension 2D or three-dimension (3D) structures to meet the required demands for supercapacitor application. 1D MXene/CNT materials are found in the form of fiber or yarn.^71^ Yu *et al.* dropped MXene solution on CNT scaffold and after drying the MXene/CNT ink, it is peeled off and scrolled into a helical fiber by Archimedean spirals.^72^ 2D MXene/CNT hybrid material can be prepared in the form of thin film, paper, nanosheets or coating on textile substrate.^73^ Weng *et al.* utilized layer by layer method to fabricate MXene/CNT composite film. For this, they sprayed MXene/PVA suspension (positively charged particle) on CNT/PSS (negatively charged particle) to prepare the composite layer.^74^ In case of 3D structure of MXene/CNT hybrid material, it forms in foams or aerogel.^75,76^ The as-prepared MXene/CNT hybrid material with different architectures exhibits unique mechanical, electrical, low density, making this hybrid structure a potential material for supercapacitor applications. The application of MXene/CNT hybrid material's structure in supercapacitor is extensively discussed in the next section.

#### 3.1.2 Capacitive performance of 1D MXene/CNT hybrid materials

**Fig. 4:**
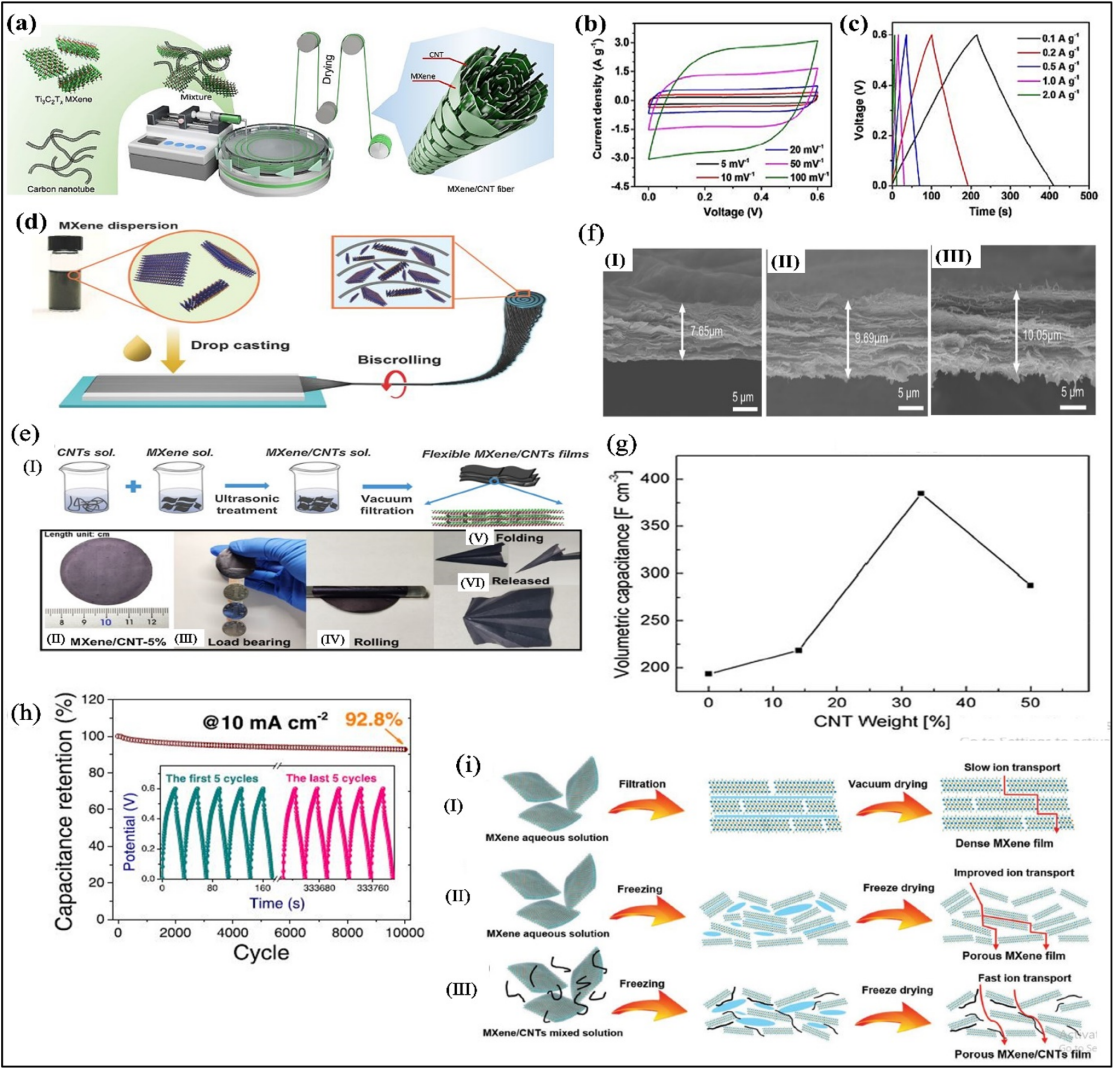
(a) Schematic illustration wet spun MXene/CNT hybrid fibers;^77^ (b) the CV curves and (c) GCD curves of symmetric supercapacitors at various scan rate and current density, respectively;^77^ (d) schematic illustration of biscrolling technique to fabricate MXene/CNT yarn;^78^ (e) (I) vacuum filtration technique to fabricate MXene/CNT technique with (II) top view of the 5 wt% mass ratio of MXene/CNT film, (III) loading test, (IV) rolling test and (V and VI) folding test of the MXene/CNT films;^79^ (f) SEM image of MXene/CNT hybrids with thickness (I) 7.65 μm, (II) 9.69 μm and (III) 10.05 μm for pure MXene, 5 wt% and 10 wt% mass ratio of MXne/CNT respectively;^79^ (g) the volumetric capacitance as a function of the CNT content;^64^ (h) cyclic performance of Ti_3_C_2_T_*x*_/CNT hybrid supercapacitor;^80^ (i) schematic illustration of electrolyte ion transportation pathways of (I) vacuum-dried dense MXene film, (II) freeze-dried porous MXene film, and (III) freeze-dried MXene/CNT film.^81^

Zhao *et al.*^77^ used wet spinning technique to fabricate MXene/CNT fibers for supercapacitors application, Fig. 4(a). Regarding the MXene/CNT wet spinning solution preparation, CNT was dispersed into sodium taurodeoxycholate (STDOC) surfactant in order to form strong hydrogen bond between CNT and MXene. The as prepared fiber not only showed enhanced mechanical (∼61 MPa) and electrical performance (∼1142 S cm^−1^) with a very low CNT content of ∼1 wt% than pure MXene but also increased the interlayer spacing of MXene from 13.5 Å to ∼17 Å. They found that with the increasing of CNT loading, the equivalent series resistance (ESR) value was reduced, revealing the successful reduction of charge transfer resistance in the 1D hybrid fiber that resulted in high specific capacitance (295 F g^−1^ at 5 mV s^−1^ in 1 M H_2_SO_4_ electrolyte solution) which can be attributed to the porosity of hybrid fibers.^77^ Furthermore, to evaluate the capacitive performance for practical applications, they woven MXene/CNT fiber electrode into the cotton fabric to construct the symmetric supercapacitor. The symmetric supercapacitor displayed rectangular and triangular shape regarding CV and GCD tests even with the increasing of scan rate and current density respectively, Fig. 4(b) and (c), proving the high energy storage capacity. In addition, it also showed high gravimetric energy density of ∼5.79 mW h g^−1^.^77^

In addition, Wang *et al.* used “Biscrolling” technique to prepare Ti_3_C_2_T_*x*_ MXene/CNT yarn, as shown in Fig. 4(d).^78^ For this, they decorated five layers of CNT sheets on a glass substrate with fixing an electric motor at the end. Then Ti_3_C_2_T_*x*_ MXene dispersion were then dropped on the CNT sheets followed by pulling by motor to obtain MXene/CNT yarn. The biscrolled yarn showed volumetric and gravimetric capacitances of 1083 F cm^−3^, 532 F g^−1^ in 3 M H_2_SO_4_ electrolyte solution. Besides, they also fabricated symmetric supercapacitor with PVA/H_2_SO_4_ gel electrolyte that demonstrated the highest energy and power density of 8.54 mW h cm^−3^ and 530 mW cm^−3^ respectively. Regarding aqueous electrolyte solution, the highest gravimetric capacitance of biscrolled yarn (532 F g^−1^ (ref. 78)) than Wet Spun Yarn (295 F g^−1^ (ref. 77)) may be attributed to the molar concentration of H_2_SO_4_ electrolyte, because enhanced concentration increases the ion conductivity of electrolyte that causes the increase of specific capacitance of supercapacitor.^4^ In a similar studies, MXene suspension was drop-casted on CNT scaffold and then MXene/CNT film was peeled off and scrolled into fiber formation, and in this helical structured fiber, MXene was wrapped into CNT corridor.^72^ CNTs maintain highly orientation in this hybrid fiber, providing high mechanical strength electrical conductivity without sacrificing plenty of spaces, contributing to the more ion transportation for enhanced capacitance.^72^

#### 3.1.3 Capacitive performance of 2D MXene/CNT hybrid materials

Besides the 1D MXene/CNT fiber structure, film like 2D MXene/CNT structure was also seen to fabricate. Chen *et al.*^79^ prepared Ti_3_C_2_T_*x*_ MXene/carbon nanotubes (CNTs) composite film by simple vacuum filtration technique and the prepared composite film was folded into various shapes with load bearing capacity, as demonstrated in Fig. 4(e). In addition, they found that increasing CNT loading enhanced the interlayer structure, Fig. 4(f), however, the increased CNT loading in the hybrid film lowered the capacitance from 300 F g^−1^ (5% loading of CNT) to 265 F g^−1^ (10% loading of CNT) at the current density of 1 A g^−1^.^79^ Moreover, GCD analysis of different CNT loading (0%, 1%, 5% and 10%) revealed that the MXene/CNT-5% showed the longest time of charge–discharge process among the other composition of hybrid films, confirming the largest capacitance. Although, the addition of CNT enhances the restacking problems of MXenes with high capacitance performance by increasing the interlayer spaces, more addition of CNT may reduce the capacitance performance of supercapacitors because CNT has lower capacitance and conductivity than MXenes.^79^ Similar result was also found with the increasing of CNT loading by Yan *et al.*^64^ They mixed d-Ti_3_C_2_ and CNT in different ratios followed by filtration in order to get 2D Ti_3_C_2_/CNT hybrid materials. In case of electrochemical performance in an alkaline electrolyte solution, they noticed that with the increasing of CNT content, the volumetric capacitance was increased gradually which began to decrease with further increasing of CNT content, as shown in Fig. 4(g).^64^ Although the capacitance was decreased with more CNT loading, the Ti_3_C_2_/CNT hybrid's capacitance performance of different ratios was still better than pure MXene which proved the increasing of distance between Ti_3_C_2_ sheets along with the overcome of Ti_3_C_2_ sheets aggregation.^64^ So, it can be conferred that the porous structure of MXene/CNT hybrid are the primary reason to enhance the capacitive performance. To introduce the more of porous structure in MXene/CNT hybrids, Li *et al.*^82^ followed new strategy where they added NaOH into Ti_3_C_2_T_*x*_ MXene/CNTs mixture. The introduction of NaOH disrupted the electrostatic repulsion between the MXene sheets which causes the MXene flakes to be wrinkled and flocculated, forming Ti_3_C_2_T_*x*_ MXene/CNTs flocs which was further vacuum filtrated into Ti_3_C_2_T_*x*_ MXene/CNTs film. This hybrid film overcomes the as usual dense stacking of 2D film by forming a more porous structure. Moreover, for better electrochemical performance, the alkali induced Ti_3_C_2_T_*x*_ MXene/CNT film was annealed at 400 °C to eliminate the fluorine and hydroxyl terminations in order to promote the transport of electrolyte ions. The as prepared hybrid film displayed the specific capacitance of 336.2 F g^−1^ which was better than alkali induced MXene film (280.9 F g^−1^) at the high current density of 1000 A g^−1^ that can be attributed to the more developed porous structure of alkali induced Ti_3_C_2_T_*x*_ MXene/CNT film than the alkali induced MXene film.^82^ Moreover, a new film of Co@N-CNT/Ti_3_C_2_T_*x*_ MXenes was also developed as an electrode material to fabricate a flexible solid-state symmetric supercapacitor where PA/LiCl gel was used as an electrolyte.^83^ This symmetrical supercapacitor displayed excellent cycling stability (85 000 cycles) and coulombic efficiency (99.7%) for their high surface area and pseudocapacitance.^83^

Besides the widely used Ti_3_C_2_T_*x*_-MXene, Nb_2_CT_*x*_ MXene hybrid with MWCNTs was also used by Xiao *et al.*^84^ Here, the lower conductivity of Nb_2_CT_*x*_ than Ti_3_C_2_T_*x*_ was improved by introducing MWCNT with the Nb_2_CT_*x*_-MXene. In addition, it has been found that the specific capacitance of Nb_2_CT_*x*_/MWCNT and pure Nb_2_CT_*x*_ was 202 F g^−1^ and 186 F g^−1^ at 2 mV s^−1^ in three electrode system where 1 M H_2_SO_4_ as used as an electrolyte. This significant capacitive performance of Nb_2_CT_*x*_/MWCNT were mainly derived by introducing MWCNT as a conductive bridge.^84^

#### 3.1.4 Capacitive performance of 3D MXene/CNT hybrid materials

To achieve the highest capacitance, Yang *et al.*^80^ prepared honeycomb like Ti_3_C_2_T_*x*_@CNT hybrid sponges by electrochemical deposition, to obtain high speed ion exchange with gravimetric capacitance of 468 F g^−1^ at 10 mV s^−1^. Moreover, the prepared Ti_3_C_2_T_*x*_@CNT-based symmetric supercapacitor offered 92.8% retention after 10 000 cycles of the charge–discharge process 10 mA cm^−2^, as depicted in Fig. 4(h).^80^ The highest capacitance of 3D like MXene/CNT honeycomb sponges may be attributed to the formation of more porous structure in spongy film^80^ that results in providing more opening path for electrolyte ion exchange. The effect of this porous structure of MXene/CNT hybrids on supercapacitor applications are investigated by Zhang *et al.*^81^ They fabricated three different MXene-based films namely, a densely packed Ti_3_C_2_T_*x*_ film (D-MF) by vacuum filtration, a porous Ti_3_C_2_T_*x*_ film by freeze-drying (3D-PMF), and a porous Ti_3_C_2_T_*x*_/CNT film by freeze-drying (3D-PMCF). Furthermore, they used these films to create symmetric supercapacitors (SSCs).^81^ They observed that 3D-PMCF had the highest area of the cyclic voltammetry (CV) curve compared with D-MF and 3D-PMF, indicating the highest specific capacitance (about 375 F g^−1^). This capacitance is attributed to the bigger pore volume (0.103 cm^3^ g^−1^) of 3D-PMCF than those of the other two samples (D-MF: 0.01 cm^3^ g; 3D-PMF: 0.065 cm^3^ g^−1^), resulting in improved ion accessibility. Here, CNT acts as the spacer to increase the pore volume of PMCF than PMF and D-MF samples and for increasing porous structure, the ion transportation is fast in case of 3D-PMCF than the other samples, as demonstrated in Fig. 4(i).^81^ Moreover, a new 3D structure of Ti_3_C_2_T_*x*_-MXene/CNT hybrid was developed by Gao *et al.*^85^ Regarding this, at first, they prepared a new knotted CNT which was then dispersed in CTAB solution. After that MXene-knotted CNT composite electrodes were prepared by a self-assembly process which was further used for investigating the electrochemical performance in an organic electrolyte for maximizing ion accessibility. They found that MXene-knotted CNT hybrids showed high capacitance, up to 130 F g^−1^ (276 F cm^−3^) in organic electrolytes with a capacitance retention of ∼56% at scan rates from 10 mV s^−1^ to 10 V s^−1^. For comparison, they also prepared Ti_3_C_2_T_*x*_-MXene/non-knotted MWCNT 2D structure, however, this 2D structure only displayed a capacitance retention of 39% from 10 to 500 mV s^−1^.^85^ This proves that the 3D structure design allows more electrolyte ion accessibility than 2D structure which means 3D like MXene/CNT possess more capacitive performance. An overall comparison of preparation, structure and electrochemical performance of MXene/CNT is shown in Table 2.

**Table 2:** A comparison of electrochemical performance of MXene hybrids

Hybrid materials^a^	Preparation method & structure	Electrolyte	Optimum A. C.^a^ (F cm^−2^)	Optimum G. C.^a^ (F g^−1^)	Optimum V. C.^a^ (F cm^−3^)	C. R.^a^	Ref.
Ti_3_C_2_T_*x*_/CNT	Wet spun, fiber (1D)	1 M H_2_SO_4_	—	∼295	—	—	77
		PVA/H_2_SO_4_				83% after 5000 cycles	
				33			
MXene/CNT	Helical yarn (1D)	3 M H_2_SO_4_	3.188	532	1083	—	78
Ti_3_C_2_T_*x*_/CNT/MnO_2_	Hydrothermal and coating, composite fiber (1D)	1 M Na_2_SO_4_	—	181.8	—	91% after 5000 cycles	71
Ti_3_C_2_T_*x*_/CNT	Drop casting and scrolling, helical fiber (1D)	PVA/LiCl	—	—	22.7	84% at current density of 1A cm^−3^	72
		6 M LiCl			Approximately 90	95% at 1 A cm^−3^	
Ti_3_C_2_T_*x*_/CNT	Vacuum filtration, composite film (2D)	1 M H_2_SO_4_	—	300	—	92% after 10 000 cycles	79
Ti_3_C_2_T_*x*_/SWCNT	Vacuum filtration of sandwiched hybrids & composite paper (2D)	1 M MgSO_4_	—	—	390	No degradation after 10 000	86
	Vacuum filtration of random mixed hybrids & composite paper (2D)				300		
Ti_3_C_2_T_*x*_/MWCNT	Vacuum filtration of sandwiched hybrids, composite paper (2D)		—	—	321	—	
	Vacuum filtration of random mixed hybrids, composite paper (2D)				366		
Ti_3_C_2_T_*x*_/CNT	*In situ* growth, composite material (2D)	3 M H_2_SO_4_	—	299.52	—	84.2% after 10 000 cycles	87
Alkali induced Ti_3_C_2_T_*x*_/CNT	Vacuum filtration, composite film (2D)	3 M H_2_SO_4_	—	401.4	—	99.0% after 20 000 cycles	82
Ti_3_C_2_T_*x*_/CNT	Focused ion beam, hybrid composite film (2D)	PVA/H_2_SO_4_	0.317	—	—	—	88
Ti_3_C_2_T_*x*_/SCNT	Self-assembly, composite film (2D)	1 M KOH	0.22	—	314	95% after 10 000 cycles	89
Nb_2_CT_*x*_/CNT	MXene/CNT slurry coating on carbon paper, (2D)	1 M H_2_SO_4_	—	202	—	80.3% after 5000 cycles	84
Ti_3_C_2_T_*x*_/CNT	Layer-by-layer assembly, composite film (2D)	0.1 ml H_2_SO_4_/PVA gel	61.38	—	87.68	67.2% at current density of 5 mA cm^−2^	90
MXene/CNT/MnO_2_	Vacuum filtration assisted layer by layer strategy, composite film (2D)	1 M Na_2_SO_4_	—	221	—	Good retention during 10 000 cycles	91
Ti_3_C_2_T_*x*_/CNT	Dip coating, composite film (2D)	0.5 M Na_2_SO_4_	2.26	56.6	—	94.3% after 1000 cycles	92
Ti_3_C_2_T_*x*_/CNT	Freeze drying, porous composite film (3D)	3 M H_2_SO_4_	—	375.0	—	95.9% after 10 000 cycles	81
	Freeze drying, porous MXene film (3D)			323.3		—	
	Vacuum filtration, MXene dense film (2D)			286.8		—	
Ti_3_C_2_T_*x*_/knotted CNT	Self-assembly, MXene-knotted CNT structure (3D)	1 M EMIM-TFSI/ACN	—	130	276	Almost no decay after 10 000 cycles	85
Ti_3_C_2_T_*x*_/CNT	Electrophoretic deposition, sponge (3D)	6 M KOH	0.661	468	—	92.8% after 10 000 cycles	80

a A. C. = Areal Capacitance, G. C. = Gravimetric Capacitance, V. C. = Volumetric Capacitance, C. R. = Capacitance Retention, CNT = Carbon Nanotube, MWCNT = Multiwall Carbon Nanotube, SWCNT = Single Wall Carbon Nanotube.

### 3.2 MXene/PPy

The use of conductive polymer like polypyrrole with the notable 2D MXene structure has opened a new era for the fabrication of wearable, flexible, lightweight, and portable devices. Due to the surface termination group of M_*n*+1_X_*n*_T_*x*_, where T_*x*_ represents –O, –OH, and/or –F terminating groups, MXene exhibits superior reinforcing properties towards the conducting polymers.^93,94^ As MXene and conducting polymer exhibit excellent interfacial bonding, the hybrid material of MXene/conductive polymer offers significant advantages ranging from versatility, compatibility and high performance products. For instance, polypyrrole is a conductive polymer that is widely used for preparing energy storage devices.^95^ Intercalation of polypyrrole with MXene solves the degradation problem of MXene in the presence of water and oxygen,^96^ attracting scientists to produce novel MXene/PPy material for next generation wearable and flexible supercapacitor-based devices. In addition, the intercalation of PPy can expand the interlayer spaces of MXene with porous structure which may offer an excellent transmission of electrolyte ion during charge/discharge cycles.^97,98^ Due to the increasing of interlayer spacing and strong interfacial bonding between polypyrrole and MXene materials, an ion transfer path is created,^99^ resulting in the highest capacitance. In this section, the preparation process and application of MXene/PPy hybrids are described elaborately.

#### 3.2.1 Preparation process of MXene/PPy hybrid materials

During the preparation of MXene/PPy hybrids, the N–H group of polypyrrole and the terminating groups of MXene forms strong hydrogen bond, ensuring the deposition order of polypyrrole in the MXene structure for which ion transport pathways are created for fast charge storage.^94^ Different techniques were reported to prepare MXene/PPy hybridized material such as *in situ* polymerization, self-assembly and electro polymerization. Tong *et al.*^97^ fabricated Ti_3_C_2_T_*x*_/PPy hybrid films using the *in situ* polymerization technique. In this work, 5 mg ml^−1^ of Ti_3_C_2_T_*x*_ and 80 μL pyrrole (monomer) solution were mixed under mechanical agitation and then placed into an ice bath. Then 15 mg ml^−1^ of APS (oxidant) solution was added dropwise into the above solution to initiate the polymerization. Chen *et al.*^98^ also followed the *in situ* polymerization technique to prepare MXene/PPy nanocomposite film. They prepared Ti_3_C_2_T_*x*_ solution by adding HCL into pyrrole solution followed by stirring at 2 °C. Next, APS was added into the solution mixture to initiate the polymerization. Another technique of the *in situ* polymerization involves the oxidant free polymerization.^99^ In this case, the terminating group –OH of Ti_3_C_2_T_*x*_ with acidic nature promotes the proton transfer from –OH group to pyrrole monomer initiating the polymerization and form hydrogen bond to get freestanding MXene/PPy nanocomposite.^100^ In addition to the *in situ* polymerization of PPy and MXene, it was reported that electrochemical deposition is also applied to fabricate the MXene/PPy hybrid composite film.^101^

#### 3.2.2 Capacitive performance of 1D MXene/PPy hybrid materials

**Fig. 5:**
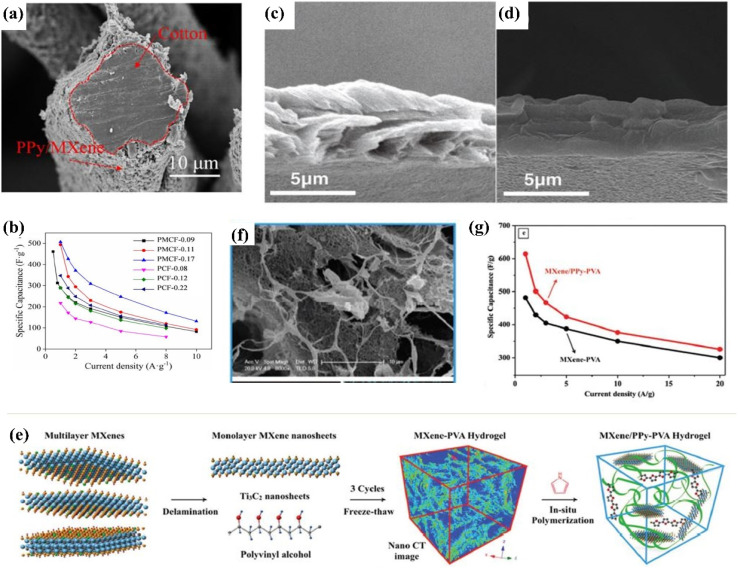
(a) Cross section of MXene/PPy wrapped around the cotton fiber;^102^ (b) specific capacitance of PPy@cotton fiber and (PPy/MXene)@cotton fiber with different mass loading of electrochemically active substance;^102^ (c and d) cross-section of porous PPy/l-Ti_3_C_2_ film and dense PPy film respectively;^103^ (e) schematic diagram of MXene/PPy–PVA hydrogel fabrication process;^104^ (f) SEM image MXene/PPy–PVA hydrogel with porous structure;^104^ (g) comparison of specific capacitance between MXene/PPy–PVA and MXene/PVA hydrogel at different current density.^104^

1D fiber electrode can meet the requirement of wearable electronic device by making the flexible textile based supercapacitor with fast charging/discharging and long cycle life. The fiber electrode can be easily integrated into textiles by weaving or knitting, facilitating the preparation of textile based supercapacitor. Yang *et al.*^102^ prepared a porous core–shell PPy/Ti_3_C_2_T_*x*_ MXene@cotton fiber (PMCF) electrode by *in situ* polymerization technique to investigate the electrochemical performance in order to use PMCF as a flexible energy-storage device in the future. Fig. 5(a) shows that the MXene/PPy was wrapped around the cotton yarns which formed a core (cotton fiber) and shell (MXene/PPy) shell structure. This core–shell structure made a porous model, ensuring the electrolyte ion transfer pathways to enhance the capacitive performance. It was found that with the increasing of PPy/MXene loading on cotton fiber, the specific capacitance was increased which was even better than the individual PPy coated cotton fiber, as demonstrated in Fig. 5(b), and this enhanced capacitance can be attributed to the formation of a more porous structure with the increasing of electroactive materials loading.^102^ Moreover, the remarkable conductivity and mechanical strength of the hybrid materials material make it a promising candidate for future flexible energy storage devices.^102^

#### 3.2.3 Capacitive performance of 2D MXene/PPy hybrid materials

Free-standing 2D composite film is a widely used morphological structure for supercapacitor applications. Zhu *et al.*^103^ followed the electrophoretic deposition to prepare freestanding PPy/layered Ti_3_C_2_ film which formed a porous structure, Fig. 5(c) and (d), due to the existence of MXene material. This porous structure enhanced the electrolyte ion transfer pathway during the charge–discharge process, benefitting high capacitive performance.^103^ It had been found that the capacity of PPy/layered Ti_3_C_2_ film reached to 406 F cm^−3^ which was 30% more than the pure PPy free-standing film (about 300 F cm^−3^), confirming the formation of a more porous structure in PPy/layered Ti_3_C_2_ film than the pure PPy film.^103^ Moreover, a solid-state supercapacitor was also fabricated by using PPy/layered Ti_3_C_2_ film which demonstrated an excellent capacitance of up to 35 mF cm^−2^ and perfect cycling stability.^103^ Besides, the electrodeposition techniques, *in situ* polymerization technique was also adopted by Boota *et al.*^105^ However, in-stead of using any oxidant, they took the advantage of strong acidic character of MXene as well as hydrogen bond between MXene and pyrrole, that may assist in formation of the aligned polymerized chains. After self-assembled polymerization process, vacuum filtration was used to get free-standing film.^105^ The as fabricated PPy/Ti_3_C_2_T_*x*_ exhibited higher volumetric capacitance of ≈1000 F cm^−3^ and capacitance retention of 92% after 25 000 cycles which was due to the hydrogen bonding, increased interlayer space of composite film and surface redox processes of the PPy and MXene.^105^ Although the MXene/PPy can exhibit interesting result, some drawbacks, such as time consuming in electrolyte ion transportation or filling out electrolyte gel in cell assembly still exist that can be improved by using liquid electrolytes as spacer.^106^ Fan *et al.* used an innovative strategy by addressing this challenges, where they introduced both polymerized polypyrrole (PPy) particles and ionic liquid (ILs)-based microemulsion particles as “dual spacers”, to fabricate functionalized Ti_3_C_2_-MXene composite films for high-performance and wide-temperature application in supercapacitors. Their prepared composite electrode displayed excellent rate capability between 4 °C and 50 °C as well as high gravimetric energy density of 31.2 W h kg^−1^.^106^

#### 3.2.4 Capacitive performance of 3D MXene/PPy hybrid materials

Conductive hydrogels are combined with electroactive materials within the porous network and for this reason, a superior conductive path and ion diffusion network are achieved that offers excellent capacitive performance for supercapacitor applications. In order to investigate the capacitive performance of conductive hydrogel, Zhang *et al.*^104^ prepared Ti_3_C_2_-MXene/PPy/PVA hydrogels. Regarding this, they first fabricated Ti_3_C_2_-MXene/PVA by freeze-drying method, and then by following the *in situ* polymerization technique, Ti_3_C_2_-MXene/PPy/PVA was prepared with porous structure, as shown in Fig. 5(e) and (f). Fig. 5(g) displays that MXene/PPy/PVA hydrogel offers a specific capacity of 614 F g^−1^ at 1 A g^−1^ current density which was better than the capacity (lower than 500 F g^−1^) of MXene/PVA hydrogel and even at higher temperature, the specific capacitance of MXene/PPy/PVA was higher than the MXene/PVA.^104^ This enhanced capacitance was attributed to the intrinsic conductive properties of PPy that act as a conductive bridge to connect MXene nanosheets, enhancing the electrochemical performance. Moreover, a solid-state supercapacitor was decorated by two identical MXene/PPy–PVA hydrogel electrodes with a layer of H_2_SO_4_/PVA gel electrolyte, which exhibited high capacitance (184 F g^−1^) with 83% capacitance retention over 1000 cycles.^104^ In another study, the *in situ* technique was applied, however, instead of PVA, nickel foam was used to create a conductive 3D morphology of Ti_3_C_2_T_*x*_@PPy Nanowires (NW) composite.^107^ In this study, almost similar capacitance (610 F g^−1^) and rate capability (100% after 14 000 cycles) were fond which was attributed to the PPy nanowires matrix which connected separated MXene blocks through porous structure, enabling highly ions and charges transport for high supercapacitor performance.^107^

Besides the *in situ* polymerization process, the electrochemical deposition technique was also used to fabricate 3D carambola-like structures.^108^ First, 2D Ti_3_C_2_T_*x*_-MXene was added with pyrrole monomer and then an electric field was applied.^108^ In this case, the pyrrole monomer was polymerized in the layered space of Ti_3_C_2_T_*x*_-MXene where the wide functional groups of MXene nanosheets acted as a core polymer, forming carambola like structure. When the current density was increased from 0.5 A g^−1^ to 8 A g^−1^, the as decorated carambola like MXene/PPy displayed 50% capacitance retention which was about 2.4% for pure PPy film. This excellent capacity retention was attributed to the formation 3D structure due to providing more pathways to promote the electrolyte ions. In addition, the symmetric supercapacitor decorated by 3D carambola-like MXene/PPy structure which showed a high specific capacitance of 184 F g^−1^ at a scan rate of 10 mV s^−1^ and superior capacity retention of about 86.4% after 5000 cycles.^108^ An overall comparison of preparation, structure and electrochemical performance of MXene/PPy is shown in Table 3.

**Table 3:** A comparison of electrochemical performance of MXene/PPy hybrids

Hybrid materials^a^	Preparation method and structure	Electrolyte	Optimum (A. C.)^a^ (F cm^−2^)	Optimum (G. C.)^a^ (F g^−1^)	Optimum (V. C.)^a^ (F cm^−3^)	C. R.^a^	Ref.
PPy/MXene@cotton	*In situ* polymerization, porous core–shell structure (1D)	1 M H_2_SO_4_	—	506.6	0.456	83.3% after 2000 cycles	102
Ti_3_C_2_T_*x*_/PPy	*In situ* polymerization of pyrrole, composite film (2D)	1 M H_2_SO_4_	—	437	—	78% after 1000 cycles	109
Ti_3_C_2_T_*x*_/PPy	*In situ* polymerization, composite film (2D)	0.5 M Na_2_SO_4_	2.11	52.75	—	—	110
Ti_3_C_2_T_*x*_/PPy	*In situ* polymerization of pyrrole, organ like composite (2D)	1 M Na_2_SO_4_	—	184.36	—	83.33% after 4000 cycles	111
Ti_3_C_2_/PPy	*In situ* polymerization, composite film (2D)	1 M H_2_SO_4_	—	416	1000	92% after 25 000 cycles	105
Ti_3_C_2_/PPy	Electrochemical polymerization, freestanding composite film (2D)	0.5 M H_2_SO_4_	0.203	—	406	100% after 20 000 cycles	103
		PVA/H_2_SO_4_	0.035		2.39	No decay after 10 000 cycles	
Ti_3_C_2_T_*x*_/PPy	Electrophoretic deposition and electrochemical polymerization, composite film (2D)	2 M H_2_SO_4_	0.109	—	—	96% after 10 000 cycles	101
		PVA/H_2_SO_4_	0.0867			—	
PPy/Ti_3_C_2_	*In situ* polymerization & heterostructure nanocomposite (2D)	1 M H_2_SO_4_ (3 electrode)	—	458	—	83.64% after 1000 cycles	112
		1 M H_2_SO_4_ (2 electrode)		155.61		73.68% after 4000 cycles	
Ti_3_C_2_T_*x*_/PPy	Electrostatic self-assembly and *in situ* polymerization, textile electrode (2D)	1 M Na_2_SO_4_	1.295	439	—	94.8% after 30 000 cycles	113
Ti_3_C_2_T_*x*_/PPy	Dip-dry and electrochemical deposition, textile electrode (2D)	1 M H_2_SO_4_	—	343.20	—	—	114
Ti_3_C_2_/PPy/PVA	Freeze drying and *in situ* polymerization, hydrogel (3D)	1 M H_2_SO_4_	—	614		100% over 10 000 cycles	104
		H_2_SO_4_/PVA gel	—	184	—	83% over 100 cycles	
MXene/PPy	Electrochemical polymerization & carambola-like composite (3D)	1 M H_2_SO_4_	—	416	—	86.4% after 5000 cycles	108
Ti_3_C_2_T_*x*_@PPy NW	*In situ* polymerization & porous composite structure (3D)	3 M KOH	—	610	—	100% after 14 000 cycles	107

a A. C. = Areal Capacitance, G. C. = Gravimetric Capacitance, V. C. = Volumetric Capacitance, C. R. = Capacitance Retention, PPy = Polypyrrole, PVA = Poly Vinyl Alcohol.

### 3.3 MXene/PANI hybrid

At present, as a conductive polymer, polyaniline (PANI) is widely used for various purposes such as super capacitors, electrodes, electromagnetic shielding, wearable gas sensor and human motion monitoring sensor. The intrinsic conductivity, low cost, ease of processibility, thermal and environmental stability and faradaic pseudo capacitance makes PANI an excellent substrate for supercapacitor applications.^115,116^ The choice of PANI for the supercapacitor devices has been facing flaws, for example, PANI gets stacked during the preparation of thin film. This is because in aniline monomer, lone electron pair of nitrogen atom is attracted by the benzene due to the resonance, causing electron cloud in benzene structure. Thus, it aggregates in aqueous solution and shows improper film forming properties.^117^ To improve the low dispersibility of polyaniline and get better electrochemical performance, it is necessary to disperse the polyaniline uniformly, thus the interaction of MXene with PANI can solve the low dispersibility problem. Furthermore, it also solves the restacking problem as well as increase the interlayer space of MXene,^118^ improving the surface wettability of Ti_3_C_2_ for more active sites and provide faradaic reactions, thus improving the electrochemical performance.^119^ In the coming subsection, the preparation and application of MXene/PANI in supercapacitor is discussed.

#### 3.3.1 Preparation process of MXene/PANI hybrid materials

MXene/PANI hybrids can be prepared by numerous fabrication techniques such as layer-by-layer assembly, *in situ* polymerization, electropolymerization, dip coating, hydrothermal reaction, and so many others.^120^*In situ* polymerization is a referred fabrication method to polymerize the aniline monomer onto MXene structure either with^109^ or without the aid of an oxidant.^121^ Such preparation process of MXene/PANI may contribute higher electrical conductivity with enhanced mechanical properties. Wei *et al.*^122^ and Zhao *et al.*^123^ used *in situ* polymerization technique to prepare MXene/PANI hybrids. Zhao and other co-workers mixed Ti_3_C_2_T_*x*_/HCl and aniline solutions together and then the APS/HCl solution was added into the mixed solution to initiate the polymerization at 0–5 °C.^123^ Yun *et al.* followed layer by layer fabrication strategy with a glass substrate that was first immersed into polyaniline nanofiber (PNF) and then dipped into MXene solution.^124^ This process was repeated several times until the desired layer obtained. Yin *et al.* also used layer by layer polymerization technique to fabricate MXene/PANI hybrids.^125^ Furthermore, Jia *et al.*^126^ used dip coating process to prepare MXene/PANI based hybrids.

**Fig. 6:**
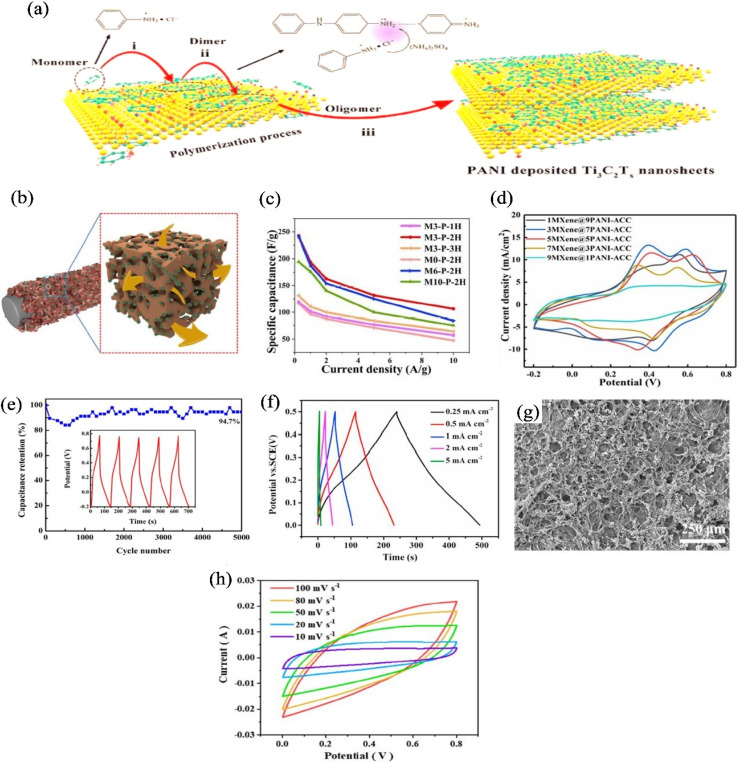
(a) Schematic illustration of MXene/PANI polymerization mechanism,^129^ (b) ion migration of MXene/PANI coated activated carbon cloth;^130^ (c) capacitive performance of CF@MXene/PANI composite fiber with different mass loading of MXene and polymerization time;^131^ (d) CV curves of MXene/PANI coated activated carbon cloth electrode with different mass ratio of MXene and PANI;^130^ (e) cyclic performance of Ti_3_C_2_/PANI-NT electrodes (5000 cycles at 1 A g^−1^), the inset exhibits the GCD curves of the last five charge–discharge cycles;^132^ (f) GCD curve of organ-like Ti_3_C_2_ MXenes/polyaniline hybrids at different current density with almost triangular characteristics;^133^ (g) SEM image of PANI@Ti_3_C_2_T_*x*_/PVA hydrogel with sponge structure;^134^ (h) cyclic voltammetry (CV) curve of PANI@Ti_3_C_2_T_*x*_/PVA with rectangular shape and broad redox peak.^134^

During the preparation process of MXene/PANI hybrids, polyaniline acts as a conductive bridge for linking adjacent layers of MXene together, accelerating the charge transfer among different MXene layers.^127^ The anchored PANI on the MXene surface can provide many active sites for rapidly transferring electrolyte ions.^128^ In case of MXene/PANI hybrids, positively charged aniline and negatively charged functional groups (*e.g.*, Ti–OH– and Ti–F–) on the surface of MXene would attract each other. The electrostatic interactions promote nanostructured PANI anchored on the surface of MXene, and then the formed PANI nanostructures prevent MXene layers from stacking and collapsing.^120^ Cai *et al.*^129^ proposed possible mechanism of polyaniline and MXene. They mentioned that cationic radicals of aniline monomer are produced during the polymerization of aniline. Negatively charged Ti_3_C_2_T_*x*_ nanosheets are able to attract positively charged radicals by electrostatic adsorption, as demonstrated in Fig. 6(a).^129^ Thus, aniline monomers can be anchored on the surface of Ti_3_C_2_T_*x*_ nanosheets, and the oxygen and hydroxyl functional groups act as anchored sites.^129^

#### 3.3.2 Capacitive performance of 1D MXene/PANI hybrid materials

1D like fiber, yarn or wire like electrodes prepared with electroactive materials, offer high flexibility with superior capacitive performance. Liu *et al.*^130^ prepared MXene/PANI/carbon fiber hybrids where 1D carbon fibers were covered with Ti_3_C_2_T_*x*_-MXene/PANI by drop coating method and MXene/PANI were uniformly packed on the fiber surface. The uniformly packed Ti_3_C_2_T_*x*_/PANI hierarchical structure not only solved the agglomeration of PANI and self-stacking of MXene nanosheets but also provided porous structure that facilitated the electrolyte ion migration during charge–discharge process, as shown in Fig. 6(b), resulting in an excellent charge storage performance. For this porous conductive materials on the fiber surface, MXene/PANI/carbon fiber demonstrated a high areal capacitance of 1347 mF cm^−2^ at a constant current density of 1 mA cm^−2^ along with 81% capacity retention after 5000 cycles at 20 mA cm^−2^.^130^ Similarly, in another study, instead of drop coating procedure, it has been found that carbon fiber@Ti_3_C_2_T_*x*_ MXene/PANI fiber electrodes were prepared by Cheng *et al.* by following the *in situ* “co-growth” technique which offered 3D porous structure on the fiber surface.^131^ Cheng *et al.* found that with the increasing of polymerization time of aniline monomer, denser and compact sized particles were formed on the fiber surface and also by increasing the MXene content, agglomeration was found; which negatively affected the capacitance performance of the electrode materials, as shown in Fig. 6(c).^131^ Hence, they optimized the electroactive materials with 30 mg MXene and 2 h polymerization time which showed the capacitance of 193.75 F g^−1^ at current density of 1 A g^−1^, and the 89% capacitance retention was after 2000 charge–discharge cycles.^131^ Almost similar concept has also been found in a study,^130^ where Liu *et al.* optimized the MXene/PANI mass ratio with 3 : 7 *in lieu* of using 1 : 9, 5 : 5, 7 : 3, 9 : 1 mass ratio, because 3MXene@7PANI carbon cloth exhibited the largest peak current and integration area than other composition, as shown in Fig. 6(d), confirming the highest capacity.^130^ From the above discussion, it can be conferred that excess amount of MXene or PANI can impede the individual capacitive performance which may not allow us the purpose of using MXene/PANI composites; therefore, before using MXene/PANI based composite fiber in supercapacitor applications, the content of MXene/PANI must be optimized.

#### 3.3.3 Capacitive performance of 2D MXene/PANI hybrid materials

Wu *et al.*^132^ fabricated Ti_3_C_2_/PANI-nanotube (NT) electrodes following the *in situ* polymerization of aniline monomer on the Ti_3_C_2_ surface using malic acid and tartaric acid as the organic proton acid and ammonium persulfate as the oxidant. They noticed that the tube structured of polyaniline causes the MXene/PANI NT prepared by malic acid, which was denoted as Ti_3_C_2_/PANI-NT-1, showed higher capacitive performance than the tartaric acid prepared electrode, which was denoted as Ti_3_C_2_/PANI-NT-2. The highest capacitive performance was attributed to the less –OH group of malic acid than tartaric acid, causing the lesser pore volume and specific capacitance of Ti_3_C_2_/PANI-NT-2. Finally, in a typical three-electrode system with 1 M H_2_SO_4_ aqueous electrolyte, Ti_3_C_2_/PANI-NT-1 offered high specific capacitance (596.6 F g^−1^) at 0.1 of A g^−1^ and excellent cyclic stability (94.7%) measured by the GCD test at 0.1 A g^−1^, because of providing more ion transport channels by PANI-NTs. The long term cyclic stability,displayed in Fig. 6(e), revealed the enhanced pseudocapacitance contribution of PANI due to the cancelation of the swelling and shrinkage.^132,135^ In addition to the *in situ* polymerization method, electrochemical deposition technique was also applied, where amino functionalized Ti_3_C_2_ covalently bonded with amine nitrogen of PANI chains, ensuring faster ion diffusion path.^133^ The as-prepared hybrids showed triangular curve of GCD at various current density, as demonstrated in Fig. 6(f), confirming good electrochemical behavior with reversible characteristics of an idle supercapacitor to fabricate novel MXene/PANI hybrids. Besides the preparation of flexible thin film of MXene/PANI electrodes, wearable supercapacitor was also prepared by using MXene and PANI electroactive materials with cotton fabric.^136^ It has been found that *in situ* polymerization of aniline monomer with cotton fabric displayed lower areal capacitance (214.3 mF cm^−2^ at 1 mA cm^−2^) than the MXene/cotton and MXene/PANI@cotton electrode which was 471.3 mF cm^−2^ and 1027.5 mF cm^−2^.^136^ The enhanced capacitance of MXene/PANI modified fabric was attributed to the reducing ion diffusion pathways by MXene and providing enhanced electroactive surface by PANI.^136^

#### 3.3.4 Capacitive performance of 3D MXene/PANI hybrid materials

Introducing interlayer spacer with 3D networks creates porous structure as well as facilitates more reactive sites, thus solving the restacking problem of MXene with enhanced electrochemical performance.^137,138^ Introducing PANI with various formation, such as polyaniline nanotubes,^139^ polyaniline nanoribbons,^140^ and polyaniline nanofibers (PANINFs),^141^ as an interlayer spacer, could provide the active sites on the surface as well as the transport of electrolyte ions. Li *et al.* prepared Ti_3_C_2_T_*x*_ MXene/PANI hybrid materials, where 3D constructive network by introducing PANI nanofibers into MXene layers increased the charge transfer among different MXene layers, acting as a conductive bridge between the adjacent layers of MXene.^127^ The as prepared electrode exhibited high specific capacity of 563 F g^−1^ at 0.5 A g^−1^ and a high capacitance retention of 84.72%.^127^ Regarding the PANI nanofibers/MXene hybrids, positively charged PANINFs and the negatively charged Ti_3_C_2_T_*x*_ nanosheets are interacted with each other by electrostatic interaction and hydrogen bonding, providing abundant accessible sites and facilitate the diffusion of ions. Cao *et al.*^134^ prepared a 3D PANI@Ti_3_C_2_T_*x*_/PVA sponge structure, as displayed in Fig. 6(g). In this structure, the –OH group of PVA and –O, –OH and-F polar groups of Ti_3_C_2_T_*x*_ were interconnected by electrostatic attraction, and further PANI was *in situ* polymerized onto the surface of Ti_3_C_2_T_*x*_/PVA. The introduction of PVA into Ti_3_C_2_T_*x*_ layer *via* sol–gel and freeze dried process creates the porous sponge template and the later inclusion of PANI, further improves the pore utilization rate of the porous sponge with enhanced specific capacitance of the electrode material.^134^ The as fabricated PANI@Ti_3_C_2_T_*x*_/PVA hybrids was further used to prepare a flexible symmetric supercapacitor which showed both rectangular shape and redox peaks, indicating both the double layer capacitance and the pseudocapacitance, as illustrated in Fig. 6(h).^134^ An overall comparison of preparation, structure and electrochemical performance of MXene/PANI is shown in Table 4.

**Table 4:** A comparison of electrochemical performance of MXene/PANI hybrids

Hybrid materials^a^	Preparation method & structure	Electrolyte	Optimum A. C.^a^ (F cm^−2^)	Optimum G. C.^a^ (F g^−1^)	Optimum V. C.^a^ (F cm^−3^)	C. R.^a^	Ref.
Ti_3_C_2_T_*x*_/PANI/carbon fiber	Drop coating & hierarchical structures (1D)	1 M H_2_SO_4_	1.347	—	—	81% after 5000 cycles	130
CF@Ti_3_C_2_T_*x*_/PANI	*In situ* “co-growth” & 1D fiber with 3D coating layer.	1 M H_2_SO_4_	—	193.75	—	89% after 2000 cycles	131
Ti_3_C_2_T_*x*_/PANI	Oxidant free *in situ* polymerization, freestanding hybrid film (2D)	3 M H_2_SO_4_	—	503	1682	98.3% after 10 000 cycles	118
Ti_3_C_2_/PANI-nanotube	*In situ* polymerization, composite film (2D)	1 M H_2_SO_4_	—	596.6	—	94.7% after 5000 cycles	132
Ti_3_C_2_T_*x*_/PANI	Chemical oxidative polymerization, composite film (2D)	1 M H_2_SO_4_	—	556.2	—	91.6% after 5000 cycles	142
PANI/Ti_3_C_2_T_*x*_	Self-assembly strategy, nanohybrid film (2D)	1 M H_2_SO_4_	—	462	—	84.5% after 5000 cycles	143
Graphene decorated Ti_3_C_2_T_*x*_/PANI	*In situ* oxidative polymerization, composite film (2D)	1 M H_2_SO_4_	—	452	606	80.4% after 5000 cycles	144
Ti_3_C_2_/PANI	Electrochemical polymerization, organ like composite (2D)	0.5 M H_2_SO_4_	0.228	—	—	85% after 1000 cycles	133
PANI/Ti_3_C_2_	*In situ* polymerization, composite film (2D)	1 M Na_2_SO_4_	—	164	—	96% after 3000 cycles	119
Ti_3_C_2_T_*x*_/PANI	Electrostatic self-assembly, porous sandwich structured (3D)	—	0.959	645.7	—	98% after 5000 cycles	145
PANI@Ti_3_C_2_T_*x*_/PVA	Sol gel and *in situ* polymerization porous sponge structure (3D)	PVA/H_2_SO_4_	0.103	—	—	99% after 10 000 cycles	134
Ti_3_C_2_T_*x*_/PANI	Hydrothermal reaction, hierarchical architecture (3D)	6 M KOH	—	563	—	95.15% after 10 000 cycles	127
Ti_3_C_2_T_*x*_/PANI	Solvent-assisted self-assembly and blade coating, composite film (2D)	1 M H_2_SO_4_	—	560	1167	97.5% after 5000 cycles	135
PANI/Ti_3_C_2_T_*x*_	Self-assembly, porous structure (3D)	3 M H_2_SO_4_	—	510	1632	85.7% from 1 to 100 A g^−1^	146

a A. C. = Areal Capacitance, G. C. = Gravimetric Capacitance, V. C. = Volumetric Capacitance and C. R. = Capacitance Retention, PANI = Polyaniline.

### 3.4 MXene/graphene hybrid

2D graphene material possesses excellent electrical, thermal and mechanical properties for which this notable material has attracted scientists' attention to fabricate supercapacitor-based devices. Furthermore, graphene has a broad operating area (2630 m^2^ g^−1^) and light weight structure, making graphene a great material to prepare supercapacitor-based devices.^147^ Although the use of graphene materials (GO and rGO) enhances the electrochemical performance, it has some shortcomings like π–π bond attraction that enhances the aggregation of individual graphene suspension for which it may surpass the use of individual graphene based material for supercapacitor applications.^148^

Fabricating MXene/graphene hybrid material can solve the aforementioned problems. During the fabrication of MXene/graphene hybrids, MXene material intercalate into the graphene sheets, thus solving the aggregation problems of graphene and the hydrophilicity of MXene can improve electrochemical performance of MXene/graphene hybrid materials. For the supercapacitor applications, graphene oxide (GO) and reduced graphene oxide (rGO), derivatives of graphene, are currently extensively used. In this section, the preparation and supercapacitors application of MXene-based graphene hybrid material are highlighted.

#### 3.4.1 Preparation of MXene/graphene hybrid materials

Fabrication of MXene/graphene-based hybrid material involves different approaches, like mechanical mixing, hydrothermal process, reduction process and self-assembly. By using the electrostatic self-assembly strategy, Yan *et al.*^149^ created MXene/rGO composites in which poly(diallyldimethylammonium chloride) modified rGO has a positive charge and the MXene nanosheet has a negative charge. They mixed modified rGO and MXene suspension by ultrasonication followed by vacuum filtration to get freestanding MXene/rGO hybrid film. Liao *et al.*^150^ prepared sulphur, nitrogen doped MXene/GO suspension followed by blade coating on polyester substrate to get MXene/GO film. The film was then treated with HI acid for 30 minutes to achieve MXene/rGO hybrid film.

**Fig. 7:**
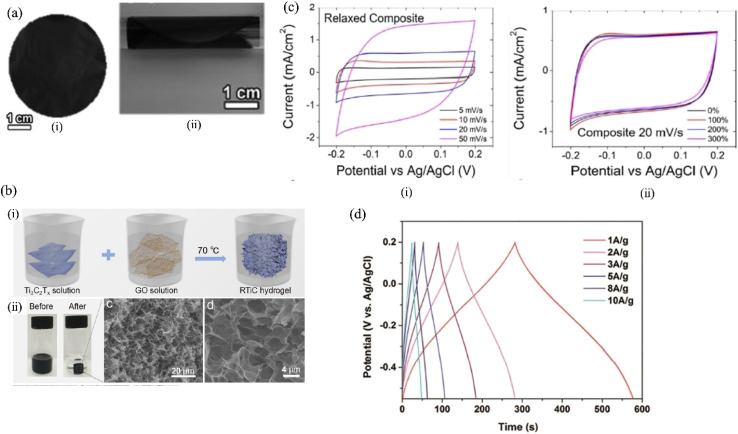
(a) Flexible (i) and freestanding (ii) MXene/rGO hybrid film,^149^ (b) (i) synthesis process of Ti_3_C_2_T_*x*_/rGO hydrogel (ii), digital and SEM image of Ti_3_C_2_T_*x*_/rGO,^151^ (c) rectangular shape of MXene/rGO (i) under relaxed state indicating double layer capacitive behavior, identical CV curves of MXene/rGO (ii) under different stretch condition indicating excellent electrochemical properties,^152^ (d) triangle like symmetrical GCD curve of lignosulphonate modified MXene/rGO hybrid,^153^ indicating the pseudocapacitive behavior.

In addition to the 2D composite film structure shown in Fig. 7(a),^149^ 3D like hydrogel of MXene/rGO hybrids was also fabricated for supercapacitor application. Through a graphene oxide (GO)– aided self-assembly technique, Chen *et al.*^151^ developed 3D macroscopic hydrogel with enhanced porous structure. Regarding this, they kept Ti_3_C_2_T_*x*_ and GO mixture solution at 70 °C under N_2_ atmosphere for 30 hours in the presence of NaHSO_3_. The as-prepared hydrogel, depicted in Fig. 7(b) was washed with DI water. Furthermore, in order to prevent MXene from oxidizing, Zhao *et al.*^154^ added ascorbic acid to the Ti_3_C_2_T_*x*_ and GO suspension that was then undergone hydrothermal treatment at 65 °C for 3 hours. After cooling down, the resultant hydrogel was dialyzed in ethanol solvent for 6 hours followed by freeze drying. The as-prepared hydrogel exhibited high-conductive 3D Ti_3_C_2_T_*x*_/rGO porous structure. Shao *et al.*^155^ and Saha *et al.*^156^ also prepared MXene/rGO gel like electrodes for supercapacitor application.

#### 3.4.2 Capacitive performance of 1D MXene/graphene hybrid materials

Yang *et al.* prepared MXene/graphene fiber *via* wet spinning technique in order to fabricate all solid state supercapacitor.^157^ Regarding the fiber preparation, first they prepared MXene/GO fiber which was treated by a mixture of HI and acetic acid in order to decorate Ti_3_C_2_ MXene/rGO fiber. For this reduction, the electrical conductivity of Ti_3_C_2_ MXene/rGO hybrid fiber increased from 21.2 S m^−1^ to 2.9 × 10^4^ S m^−1^, suggesting the potential use as flexible electrode for supercapacitor application. Further, it was found that the prepared flexible electrode with 90 wt% of Ti_3_C_2_-MXene displayed high volumetric capacitance, 586.4 F cm^−3^, and high areal capacitance, 372.2 mF cm^−2^, which was far better than net rGO fiber (7.8 mF cm^−2^ and 16.4 F cm^−3^ respectively).^157^ This superior electrochemical performance of MXene hybrid fibers was attributed to the extra redox reaction of Ti atoms.^157^ Similarly, in another study, the wet spun MXene/rGO fiber with 88 wt% of MXene also displayed higher volumetric capacitance (about 341 F cm^−3^) than pure rGO fiber (about 29 F cm^−3^).^158^ The lower capacitance of graphene fibers may be attributed to the aggregation problem of graphene while processing fibers due to not adding any additive solution. While adding MXene solution with graphene during fiber formation, significate improvements in capacitive performance has been noticed. The addition of MXene and graphene solution together not only enhances the capacitive performance but also improves the aggregation problem of graphene and the weak interlayer interaction of MXene during the fiber formation *via* solution spinning process. In addition, capacitive performance also depends on electrolyte ion transportation.

#### 3.4.3 Capacitive performance of 2D MXene/graphene hybrid materials

In the film like 2D MXene/graphene hybrid materials, generally reduced graphene oxide acted as a conductive bridge to assemble the different layers of MXene materials which improve the smooth electrolyte ion transfer process, thus supercapacitor performance of the electrode is ameliorated significantly.^159^ In a study, it has been found that graphene acted as a mechanical skeleton between the MXene nanosheets in MXene/graphene composite electrode, prepared by electrochemically exfoliated graphene (EG) and Ti_3_C_2_T_*x*_-MXene (∼200 nm) through homogenous self-assembly, that displayed electrode film thickness of 2.5 μm with interlayer spaces; therefore, electrolyte ion transportation is promoted.^160^ When Ti_3_C_2_T_*x*_/EG was used for all solid state supercapacitor, it displayed a high volumetric capacitance up to 216 F cm^−3^ at 0.1 A cm^−3^.^160^ However, having negative charge of both graphene and MXene materials during self-assembly process may not fully recover the restacking problem of 2D materials Ti_3_C_2_T_*x*_ which may be solved by electrostatic self-assembly process.^149^ Yan *et al.* fabricated MXene/graphene composite electrode by using positively charged rGO and negatively charged Ti_3_C_2_T_*x*_-MXene which demonstrated an ultrahigh electrical conductivity of 2261 S cm^−1^ with excellent volumetric capacitance, 1040 F cm^−3^ at a scan rate of 2 mV s^−1^, and a high rate capability with 61% capacitance retention at 1 V s^−1^.^149^ The enhanced electrochemical performance is achieved due to the more open structure of electrostatic self-assembled MXene/graphene composite electrode. Moreover, for creating a high pore structure, Fan and co-workers used holey graphene oxides.^161^ In addition, they annealed Ti_3_C_2_T_*x*_ to remove –F group from MXene in order to create –OH, which created more pseudocapacitive reaction. For using holey graphene oxide and annealed Ti_3_C_2_T_*x*_, the composite electrode exhibited an ultrahigh volumetric capacitance of 1445 F cm^−3^ at 2 mV s^−1^.^161^

#### 3.4.4 Capacitive performance of 3D MXene/graphene hybrid materials

For supercapacitor application, Liu *et al.*^162^ prepared Co_3_O_4_ doped 3D MXene/RGO hybrid porous aerogels *via in situ* reduction technique of GO. In this hybrid structure, rGO acted as conductive bridge and also enhanced the ion transportation to achieve high capacitance. The prepared hybrid film with porous aerogel exhibited 345 F g^−1^ capacitance at 3A g^−1^ and 85% capacitance retention after 10 000 cycles. Zhou *et al.*^152^ fabricated Ti_3_C_2_T_*x*_/rGO hybrid materials for stretchable supercapacitor applications. Their prepared electrode showed almost rectangular CV curve at relaxed state under different scan rates resulting double layer capacitive behavior. Moreover, the identical CV curve under different strains proved excellent electrochemical and structural integrity under larger strains. Fig. 7(c) shows the CV curve at relaxed state and deformation state.^152^ Ma *et al.*^153^ followed a novel strategy where they modified MXene surface with lignosulfonate, by products of the sulfite process in the wood pulping process, and they took the advantage π–π interaction between lignosulfonate and graphene to form a 3D ultrathick aerogel structure. The as-prepared aerogel structure showed highly symmetrical GCD curve, as shown in Fig. 7(d), with 386 F g^−1^ specific capacitance which indicated a high coulombic efficiency and higher capacitive behavior. The developed 3D structure of graphene incorporated MXene hybrid material can enhance the potential window due to the enhanced interlayer space and the faster ion diffusion of MXene hybrids.^163^ This may result in enhanced electrochemical performance of electrodes for supercapacitor application. In addition to the 3D structure, Xu *et al.*^164^ prepared micro-supercapacitors by MXene/rGO (EGMX) hybrid film, showing an excellent volumetric and gravimetric capacitance of 370 F cm^−3^ and 405 F g^−1^, respectively. An overall outlook of MXene/graphene hybrids in supercapacitor application is presented in Table 5.

**Table 5:** A comparison of electrochemical performance of MXene/graphene hybrids

Hybrid materials^a^	Preparation method and structure	Electrolyte	Optimum A. C.^a^ (F cm^−2^)	Optimum G. C.^a^ (F g^−1^)	Optimum V. C.^a^ (F cm^−3^)	C. R.^a^	Ref.
Ti_3_C_2_T_*x*_/EG	Self-assembly and vacuum filtration, thin film (2D)	PVA/H_3_PO_4_ gel	—	—	216 for ASSSs	85.2% after 2500 cycles for ASSSs	160
			0.00326 for MSCs		33	82% after 2500 cycles	
Ti_3_C_2_T_*x*_/rGO	Self-assembly and filtration, composite film (2D)	2 M KOH	—	154.3	—	85% after 6000 cycles	159
rGO/Ti_3_C_2_T_*x*_	GO/Ti_3_C_2_T_*x*_ solution was thermally reduced to obtain rGO/Ti_3_C_2_T_*x*_, porous composite film (2D)	6 M KOH	—	405	370	No change after 10 000 cycles	164
MXene/rGO	Wet spinning strategy, fiber structure (1D)	1 M H_2_SO_4_	0.372	—	586.40	Excellent cycling after 3000 cycles	157
Ti_3_C_2_T_*x*_/rGO	Electrostatic self-assembly, composite film (2D)	3 M H_2_SO_4_	—	335.4	1040	No degradation after 20 000 cycles	149
Ti_3_C_2_T_*x*_/rGO	Self-assembly and freeze drying, aerogel architecture (3D)	1 M H_2_SO_4_	0.346	—	—	91% after 15 000 cycles	165
Ti_3_C_2_T_*x*_/rGO	Self-assembly of MXene and holey graphene oxide, followed by an annealing, composite film (2D)	3 M H_2_SO_4_	—	438	1445	93% after 10 000 cycles	161
Ti_3_C_2_T_*x*_/rGO	*In situ* reduction and thermal annealing process, aerogel structure (3D)	6 M KOH	—	345	—	85% after 10 000 cycles	162

a A. C. = Areal Capacitance, G. C. = Gravimetric Capacitance, V. C. = Volumetric Capacitance, C. R. = Capacitance Retention, EG = Exfoliated Graphene, MSC = Micro Supercapacitor, ASSSs = All-Solid-State Supercapacitors, rGO = reduced Graphene Oxide.

### 3.5 MXene/nanocellulose hybrid

With the increasing demand for energy storage devices and the growing concern of environmental problems, natural resources have been explored extensively to fabricate supercapacitor devices. Due to the attractive properties such as large surface area, exceptional chemical structure and high porosity, nanocellulose drew a great deal of the attention of large number of scientists to develop supercapacitor devices. Furthermore, due to light the weight characteristics of nanocellulose, it can be used as a suitable substrate for the fabrication of next generation wearable supercapacitor-based devices. Although nanocellulose is an insulating material, surface modification is possible due to the abundant hydroxyl group of nanocellulose that allows it to act as a binder of active material for supercapacitor related application.^166^ In addition, the porous structure of nanocellulose allows the ions transportation through nanocellulose based electrodes, thus enhancing the electrochemical performance of supercapacitor.^167^ In spite of having great potentiality to use nanocellulose in fabricating supercapacitor, the composition of nanocellulose base electrode materials (ratio of nanocellulose and active material such as CNT, rGO and MXene) need to be optimized to get the best performance from supercapacitor devices. Among the different active materials, MXene, a newly discovered transitional materials, were used extensively for electrode materials for its high metallic conductivity that can reach up to 8000 S cm^−1^.^168,169^ However, for supercapacitor application, MXene have been suffering from restacking problem resulting in poor ion transportation. This flaw, can be solved by incorporating nanocellulose with MXene, resulting in an increase of the interlayer spacing of MXene. This facilitates the ion transportation path and further enhances the electrochemical performance of supercapacitor. In this section, the preparation process and the application of MXene/nanocellulose hybrids in supercapacitor are highlighted.

#### 3.5.1 Preparation process of MXene/nanocellulose hybrid materials

**Fig. 8:**
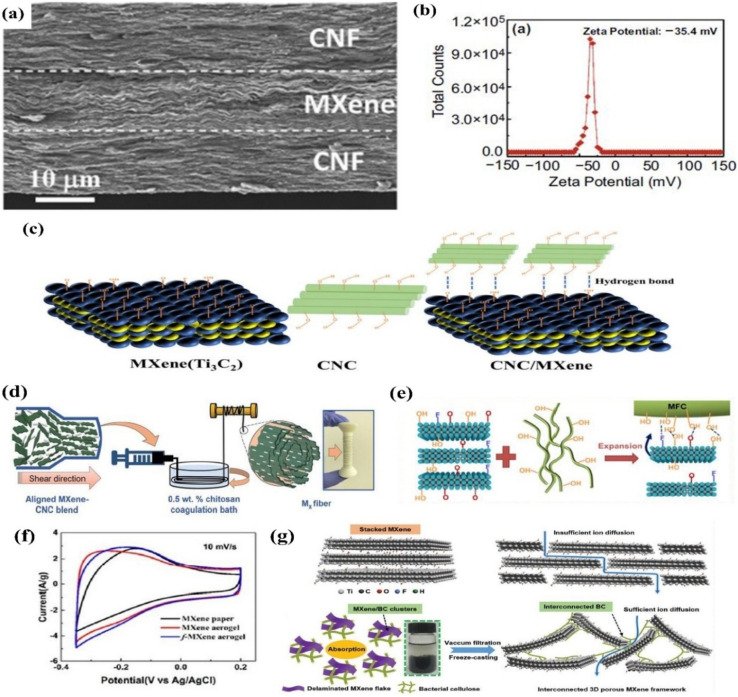
(a) SEM image of the layer by layer composition of MXene/cellulose,^173^ (b) zeta potential distribution of the MXene nanosheets dispersed in water,^174^ (c) hydrogen bonding between MXene sheets and CNC,^175^ (d) schematic illustration of wet spinning of LC-MXene/CNC fibers^176^ (e) enhanced MXene layered space when incorporating with cellulose,^177^ (f) CV curves for MXene paper, MXene aerogel, and functionalized MXene aerogel composite,^178^ (g) schematic diagram of ionic transport pathway of MXene film and 3D like MXene/bacterial cellulose composite.^179^

Different strategies were followed to fabricate MXene/nanocellulose hybrids. Feng *et al.*^170^ mixed MXene and tempo oxidized cellulose nanofiber (TOCNF) under high speed stirring on a heating stage with nitrogen condition to get MXene/TOCNF slurry. Then they made MXene/TOCNF hybrid film by blade coating on polystyrene substrate.^170^ Zhou *et al.*^171^ mixed Ti_3_C_2_T_*x*_ and tempo oxidized cellulose nanofiber by ultrasonicating and then sprayed on bacterial cellulose BC substrate using layer by layer fabrication technique. The as-prepared hybrids film possessed high mechanical strength (>250 MPa).^171^ Feng *et al.* used one-pot wet co-milling process to prepare MXene/CNF hybrid slurry.^172^ Zhou and other co-workers^173^ followed vacuum filtration fabrication technique to prepare individual Ti_3_C_2_T_*x*_ and cellulose nanofiber (CNF) suspension. Then they vacuum filtered the suspension with CNF at the bottom and top layer, as shown in Fig. 8(a), showing excellent mechanical (112.5 MPa) and electrical properties (143 S m^−1^).^173^ Song *et al.* fabricated TiC_2_/CNF flexible hybrids by mixing both Ti_3_C_2_ and CNF suspension followed by vacuum filtration.^180^ It can be observed that according to the reported research work, all reported strategies to fabricate MXene/CNF hybrids, resulted in high mechanical and electrical hybrid film.

During the exfoliation process of MXene from MAX phase by using different etching agents such as HF, NaOH, H_3_PO_4_ and LiF, abundant terminating groups (–OH, –O and –F) are usually induced. This negative terminating group of MXene can be confirmed by negative zeta potential, as it can be seen in Fig. 8b,^174^ which can form hydrogen bond with the hydroxyl group (–OH) of cellulose, providing strong bonding with the interface.^181^ Moreover, the polar groups of both MXene and cellulose possess strong interaction *via* hydrogen bonding, facilitating the solution mixture of MXene and nanocellulose to get the hybrid film. Fig. 8(c) shows the hydrogen bonding of MXene and nanocellulose.^175^

#### 3.5.2 Capacitive performance of 1D MXene/cellulose nanofiber electrode

Due to the restacking problem of MXene sheets, it exhibits lower spinnability to fabricate MXene based fibers, resulting in lower strength.^182^ Regarding this problem, adding cellulose with MXene nanosheets may offer the better solution spinnability, ensuring the strengthen of MXene/cellulose fiber with superior electrochemical performance. In a study, it has been found that Ti_3_C_2_T_*x*_-MXene-based hollow and solid core–shell fibers with regenerated cellulose (RC) by coaxial wet spinning, where RC was tough component and graphene oxide/MXene was conductive components, displayed mechanical strength of 134.7 MPa with a high conductivity of 2.37 × 10^3^ S m^−1^.^183^ In addition, to improve the spinnability with ordered structure of MXene nanosheets without binder, a new technological advancement of MXene processing is developed which is its Liquid Crystal (LC) phase, that constitute both liquid-like fluidity and crystal-like order.^184^ Zhang *et al.* showed that the LC phase of MXene fibers displayed high electrical conductivity with enhanced volumetric capacitance, ∼1265 F cm^−3^.^184^ However, binder free LC phase of MXene needs high MXene sheet size and concentration^185,186^ for imparting spinnability properties, which may make it difficult to achieve, however, S. Usman *et al.* introduced cellulose nanocrystals (CNC) into MXene sheets, offering LC phase with lower MXene sheets and concentration, ∼1 μm and ≤10 mg ml^−1^ respectively.^176^ They prepared microfibers of LC-MXene/CNC by wet spinning method, as displayed in Fig. 8(d). The improved ordering result of LC-MXene/CNC fibers resulted in high tensile strength, ∼60 MPa, high conductivity, ∼3000 S cm^−1^, and volumetric capacitance, ∼950 F cm^−3^.^176^

#### 3.5.3 Capacitive performance of 2D MXene/cellulose electrode

Due to the strong interfacial bond and the binding capabilities of cellulose, the incorporation of cellulose with MXene can enhance the mechanical properties of MXene/cellulose hybrids. Furthermore, introducing cellulose can pull and expand the MXene nanosheets, as shown in Fig. 8(e), and thus facilitating the fast ion transportation between the MXene sheets resulting in the increase of electrochemical performance.^177^ It has been found that electrostatic self-assembly between the Ti_3_C_2_T_*x*_-MXene/CNF hybrids with positively charged polyethyleneimine (PEI) showed areal capacitance of 93.9 mF cm^−2^ at a current density of 0.1 mA cm^−2^.^187^ The positively charged PEI cross-linked the negative MXene/CNF hybrids through electrostatic interaction. Therefore, hydrogen bonding between MXene and CNF as well as electrostatic interaction resulted in flexible, high strength and oriented MXene sheets that resulted in ion transportation for enhanced capacitance.^187^ In addition to facilitate the electrolyte ion transportation due to use of cellulose with MXene materials, it has also been noticed that alkalization and annealing of Ti_3_C_2_T_*x*_ improved the electrochemical performance.^188^ Besides, the widely used titanium carbide MXene for supercapacitors, Etman *et al.*^189^ used Mo_1.33_CT_*z*_ MXene to fabricate MXene/cellulose electrode by simply ultrasonicating MXene and cellulose suspension followed by vacuum filtration. The MXene/cellulose electrode displayed volumetric capacitance up to 1178 F cm^−3^ with 5 wt% cellulose content. Moreover, the composite electrode exhibited 95% capacity retention after 3000 cycles. This outstanding properties to cellulose that may provide tunneling for ion transportation, thus increasing cellulose content and enhancing the capacitance.^189^

Although nanocellulose can facilitate ion transportation between the MXene nanosheets, it may sometimes slightly decrease the electrochemical performance because of being an electrochemically inactive material. Tian *et al.* showed that 5%, 10% and 20% loading of CNF with Ti_3_C_2_T_*x*_ exhibited tensile strength of 139 MPa, 181 MPa and 340 MPa with decreasing capacitance of 369 F g^−1^, 324 F g^−1^ and 298 F g^−1^, respectively.^190^ This slight reduction of capacitance with enhanced mechanical strength does not limit the ion transportation for supercapacitor application, thus proving the practical application of CNF/Ti_3_C_2_T_*x*_ hybrid film.^190^

#### 3.5.4 Capacitive performance of 3D MXene/cellulose electrode

Preparing 3D architecture from 2D materials not only benefits from avoiding restacking problems but also gets an advantage from porous construction for electrolyte ion transportation. However, 2D materials like MXene impedes the formation of 3D structure due to the van der walls interaction between MXene nanosheets.^16^ Intercalating cellulose with MXene nanosheets enables the formation of 3D like foam, aerogel or hydrogel structure *via* template method, *in situ* foaming, freeze drying and so many others method.^182,191–193^ In a study of,^178^ Ti_3_C_2_T_*x*_-MXene composite aerogel was prepared *via* ice templating process where functionalized cellulose nanocrystal (f-CNC) served as a structural modifier and polyurethane as a cross-linker with MXene. In addition, to investigate the capacitive performance, MXene pristine paper and MXene aerogel was also prepared where it has been found that the composite aerogel showed the highest area of CV curves, demonstrating excellent electrochemical performance, as shown in Fig. 8(f) which contributed 178 F g^−1^, 201 F g^−1^, 225 F g^−1^ for pristine MXene paper, MXene aerogel, and composite aerogel respectively.^178^ The enhanced capacitive performance was obtained due to the large surface activity, excellent electrolyte interactions, and fast ion transportation.^178^ An ion transportation of pure Ti_3_C_2_T_*x*_-MXene film and 3D porous MXene framework, which constituted with Ti_3_C_2_T_*x*_-MXene and bacterial cellulose, is displayed in Fig. 8(g). When the porous 3D Ti_3_C_2_T_*x*_-MXene/bacterial cellulose was used as anode for asymmetric supercapacitor, it exhibited a high areal capacitance of 925 mF cm^−2^, a maximum energy and power density of 252 μm W h cm^−2^ and 34.02 mW cm^−2^ respectively.^179^

However, introducing additional active material with 3D MXene/cellulose hybrids can improve the mechanical and electrochemical performance with multifunctional applications which may prove the promising wearable electronics. It has been found that, Cai *et al.*^194^ introduced *in situ* grown SnS_2_ onto MXene nanosheets followed by adding CNF. By adding SnS_2_, extra H^+^ storage is achieved during the charge–discharge process which contributed specific capacitance of 171.6 F g^−1^ with high mechanical strength (78.3 MPa).^194^ Moreover, more H^+^ transport were activated by SnS_2_ under solar intensity that contributed 60% increase in capacitance under solar intensity of 1 kW m^−2^.^194^ Besides, 3D like MXene/Ag nanowires (NWs)/cellulose composite displayed a high capacitance of 505 F g^−1^ with excellent conductivity, 58 843 S m^−1^, and mechanical properties, tensile strength of 34 MPa and Young's modulus of 6 GPa.^195^

### 3.6 Challenges and future perspective

MXene is a newly discovered material with high electrical conductivity, excellent hydrophilicity characteristic due to the surface terminating groups and also it has an intrinsic capability for the fabrication of electrode materials in supercapacitor related applications. Due to the tunable surface groups of MXene and synthesis process of multiple MXene compositions, there may exist some problems for the fabrication of MXene based hybrids electrodes which should have been introduced properly. For example,

• There exists almost 20 different MXene composition and during etching of “A” element from MAX phases, tunable functional groups appeared on MXene surface. For this etching of MAX phase, different etching elements and synthesis conditions are applied, as summarized in Table 1. This synthesis procedure led to producing multilayered MXene of different compositions and wide variety of surface groups. Therefore, it is needed to further study to address the cause and solution of restacking problems of different MXene compositions. Moreover, the fabrication process of MXene hybrids should also be investigated to get the best output of MXene based electrodes. Furthermore, the most popular etching methods used to synthesis MXene, HF and LiF/HCL, are considered hazardous procedures. Therefore, due to the over growing concern of the environment, it is urgent to explore new environmentally friendly process to synthesis MXene.

• During the process of individual MXene material, aggregation problem appears due to its strong hydrophilicity that may reduce the electrochemical performance of MXene materials. For this reason, preparation of MXene based hybrid materials is the best solution in this regard. Introducing active materials with the MXene can increase the interlayer spacing and further solve the stacking problem of MXne, thus allowing the use as electrode material in supercapacitor related applications. However, the ratio of MXene and hybridized material during the preparation of electrodes should be properly investigated to guarantee high performance for supercapacitor-based application.

• Furthermore, the investigation of electrolyte performance for supercapacitor applications is needed. The capacitance property of MXene based hybrid materials largely depends on the electrolyte. There are several electrolytes used such as aqueous electrolyte, ionic electrolyte and organic electrolyte. There is different composition of MXene, and thus there are many possible MXene based hybridization compositions, so the influence of electrolyte on the performance of MXene hybridized materials should be studied elaborately for supercapacitor applications.

## 4 Conclusion

MXenes, derived from MAX phases, show great promise for supercapacitor applications due to their high conductivity, hydrophilic nature, and customizable surface chemistry; however, issues like self-restacking, oxidation, and limited ion transport restrict their full potential. By combining MXenes with carbon materials, conducting polymers, and metal oxides, researchers can significantly improve their electrochemical performance through enhanced charge storage, cyclic stability, and ion diffusion. Designing MXene-based structures in 1D, 2D, and 3D formats further optimizes electrolyte access and charge transport. Despite these advancements, challenges remain in scaling up production, achieving long-term stability, and ensuring electrolyte compatibility. Future research should prioritize developing scalable synthesis methods, innovative hybridization strategies, and environmentally friendly processing techniques to enable MXene-based supercapacitors to bridge the gap between high energy and power density, paving the way for next-generation energy storage technologies.

## Data availability

No primary research results, software or code have been included, and no new data were generated or analysed as part of this review.

## Author contributions

Tamal K. Paul: conceptualized the main idea, structured, and wrote the original manuscript; Md. Abdul Khaleque: wrote and revised of the manuscript; Md. Romzan Ali: wrote and revised the manuscript; Mohamed Aly Saad Aly: updated the main concepts and ideas, wrote, reviewed, and edited the manuscript, supervised and evaluated the overall work; Md. Sadek Bacchu: wrote sections of the manuscript; Saidur Rahman: designed, wrote, reviewed and edited sections of the manuscript; Md. Zaved Hossain Khan: contributed to the main concept, supervised and evaluated the overall concepts.

## Conflicts of interest

The authors declare that there is no conflict of interest.
